# Quantifying the strength of quorum sensing crosstalk within microbial communities

**DOI:** 10.1371/journal.pcbi.1005809

**Published:** 2017-10-19

**Authors:** Kalinga Pavan T. Silva, Prithiviraj Chellamuthu, James Q. Boedicker

**Affiliations:** 1 Department of Physics and Astronomy, University of Southern California, Los Angeles, CA, United States of America; 2 Department of Biological Sciences, University of Southern California, Los Angeles, CA, United States of America; Rice University, UNITED STATES

## Abstract

In multispecies microbial communities, the exchange of signals such as acyl-homoserine lactones (AHL) enables communication within and between species of Gram-negative bacteria. This process, commonly known as quorum sensing, aids in the regulation of genes crucial for the survival of species within heterogeneous populations of microbes. Although signal exchange was studied extensively in well-mixed environments, less is known about the consequences of crosstalk in spatially distributed mixtures of species. Here, signaling dynamics were measured in a spatially distributed system containing multiple strains utilizing homologous signaling systems. Crosstalk between strains containing the *lux*, *las* and *rhl* AHL-receptor circuits was quantified. In a distributed population of microbes, the impact of community composition on spatio-temporal dynamics was characterized and compared to simulation results using a modified reaction-diffusion model. After introducing a single term to account for crosstalk between each pair of signals, the model was able to reproduce the activation patterns observed in experiments. We quantified the robustness of signal propagation in the presence of interacting signals, finding that signaling dynamics are largely robust to interference. The ability of several wild isolates to participate in AHL-mediated signaling was investigated, revealing distinct signatures of crosstalk for each species. Our results present a route to characterize crosstalk between species and predict systems-level signaling dynamics in multispecies communities.

## Author summary

In nature, bacteria are commonly found in spatially heterogeneous mixtures. Within these environments, multiple species communicate using chemical signals, and crosstalk often governs the activities of microbial populations, including interactions with the host system, forming biofilms, and bioluminescence. Understanding such bacterial interactions is essential to control and prevent these population-level behaviors regulated by signal exchange. Additionally, quantifying bacterial crosstalk will help improve the robustness of synthetic cellular networks that utilize signal exchange.

Although cellular signaling is understood in well-mixed systems with one signal, we lack a detailed understanding of signaling in spatially distributed cellular networks or networks with multiple signals. We created an experimental system to observe and quantify microbial crosstalk between three bacterial languages. A mathematical model was implemented to predict the consequences of the exchange of multiple signals within cellular networks and good agreement between the experimental results and theoretical predictions was observed. In the mathematical model, a single parameter was sufficient to account for crosstalk between bacterial species. These experimental and theoretical tools enable us to better understand and predict how signaling influences the behavior of both natural and synthetic microbial communities.

## Introduction

Microbes communicate with each other in order to coordinate behavior and gene expression through a process known as quorum sensing. Several Gram-negative bacteria use acyl-homoserine lactones (AHLs) as a signal to communicate [1–6]. These signaling systems typically consist of a synthase, such as *luxI*, which produces a variant of AHL, and a receptor, such as *luxR*, which binds to AHLs. The receptor enacts global changes in gene expression in response to high concentrations of AHLs. Over 150 quorum sensing systems have been characterized [7,8], with most species containing one or a few signaling pathways. Each system typically produces one dominant version of AHL [7,8], and 56 different AHLs have been identified to date [7]. Variant versions of AHL involve changes in the length of the carbon chain extending from the lactone ring and chemical modifications of this carbon chain such as the addition of carbonyl groups [2,7]. Variation in the chemical structure of AHLs impacts both affinity for the receptor and the regulatory response [6–8].

Several examples of crosstalk between signaling microbes, in which signal produced from one species binds to the receptor of a second species, have been reported [1,6,9–14]. For example, *Chromobacterium violaceum*, a pathogenic Gram-negative bacteria that produces a shorter chained AHL, activates gene expression in *Vibrio harveyi*, a Gram-negative marine bacteria that produces a longer chained AHL [9]. Each such pairing of AHL and receptor inhibits or promotes the activation of gene expression to a variable degree. Multispecies communities collectively produce complex mixtures of signals and the activation of gene expression within the community is influenced by crosstalk between different AHL variants. Quantifying AHL-mediated crosstalk will help us build a predictive understanding of the signaling dynamics within heterogeneous microbial populations, potentially enabling us to control the activation of gene expression in natural and synthetic microbial communities [1,2,5,9,15–19].

**Fig 1:**
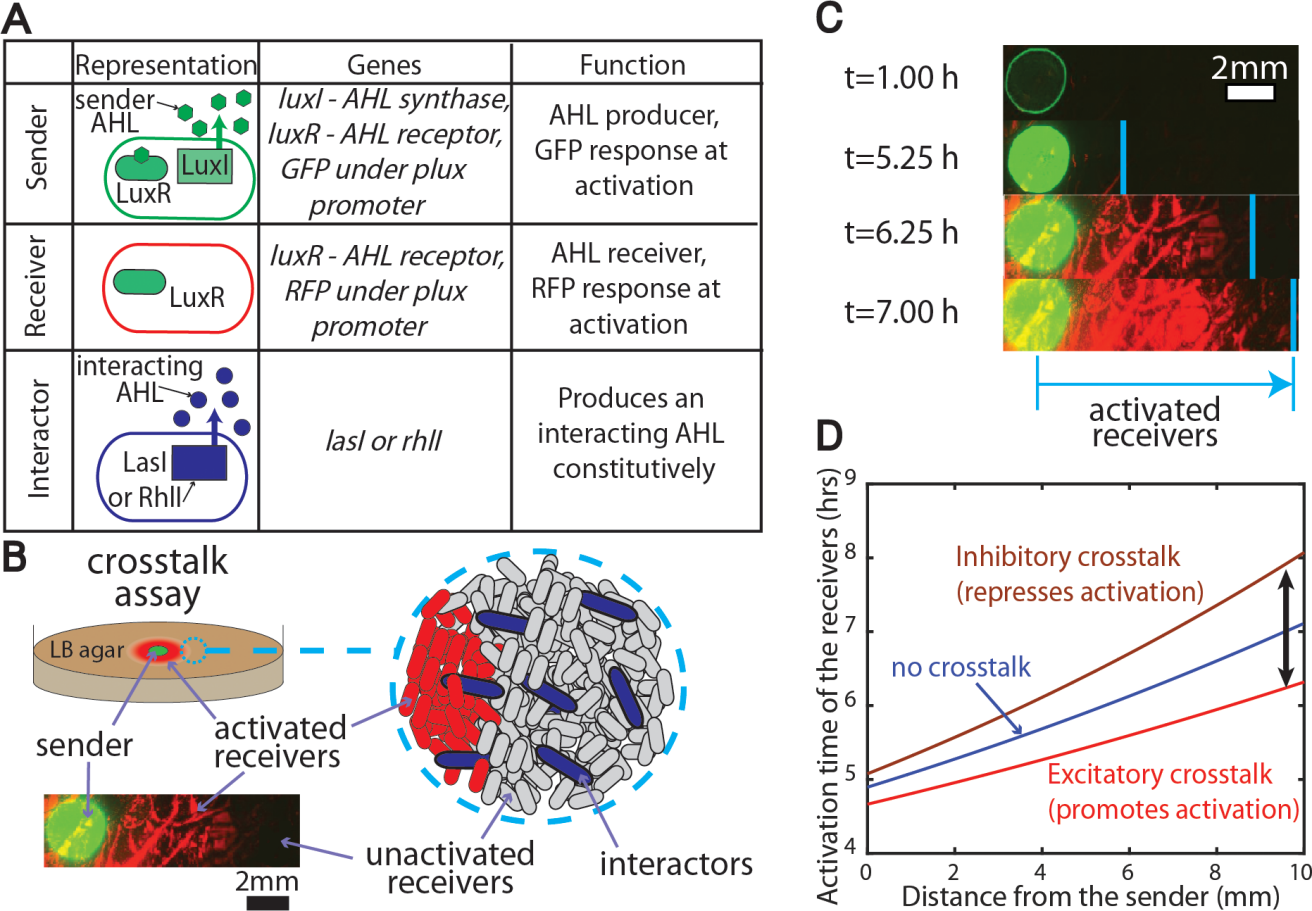
An experimental assay to quantify crosstalk between bacterial quorum sensing signals. **A.** The crosstalk assay measures the consequences of introducing a variable amount of an interactor strain into a quorum sensing network containing a sender and receiver strain. **B.** The sender strain is placed in the middle of an agar pad, surrounded by a uniform mixture of receiver and interactor strains. **C.** As the signal from the sender colony diffuses outward, the receiver strain produces a red fluorescent protein in response to a threshold level of signal. **D.** The spatial dynamics of gene expression in the receivers are compared in the presence and absence of the interactor strain. Hypothetical curves show how the interactor strain shifts the activation curve, either promoting activation (excitatory crosstalk) or repressing activation (inhibitory crosstalk).

Here we develop and implement a signaling assay using sender, receiver, and interactor strains to measure signaling dynamics in populations containing multiple AHLs, see Fig 1A. This crosstalk assay assesses the robustness of signal exchange within mixed microbial communities. Here we use a sender strain producing the AHL 3-oxo-C6 HSL and multiple interactor strains producing signals including 3-oxo-C12 HSL made by the synthase LasI and C4-HSL made by the synthase RhlI. Our work expands the scope of prior studies that focused on signal exchange in well-mixed environments, where diffusion of AHLs plays a minimal role [12,17,18,20–23]. Initial work done by Canton et al.[20] quantitatively explored the ability of multiple AHLs to bind to one receptor in a plate-reader. Wu et al. [23] used a microfluidic and flow-cytometry approach to measure the interactions between the *lux* and *las* circuits. Other studies such as McClean et al. [11] observed the response of the signaling system in *Chromoacterium violaceum* to purified variants of AHL. Although diffusion plays a role here, there was no significant quantitative work done to capture the spatial effects. In another study Dilanji et al. [24] looked at influences of a diffusive AHL wave front produced by an exogenously added chemical on agar plates. As an alternate to adding AHLs exogenously, we added a sender *E*. *coli* colony to the middle of the plate capable of synthesizing AHL molecules, as shown in Fig 1B and similar to previous experiments [25]. Our assay incorporates an interactor strain to determine the robustness of AHL signaling to crosstalk from several AHL signals, including signals produced by wild bacterial isolates.

The exchange of multiple AHLs has been reported [14,26], but these studies minimized crosstalk by using AHLs that will weakly interact with each other. Consequently, AHL crosstalk in natural contexts, where multiple signals are exchanged in spatially structured communities, is not well understood. Our study experimentally measured the effects of a neighboring interactor strain on the time scale of signaling between sender and receiver strains. The interactor strain produces a non-cognate AHL signal that influences the ability of a receiver strain to respond to the cognate AHL signal emitted from the sender strain. The consequences of such crosstalk were examined over length scales much larger than individual cells. A mathematical model was derived and compared to experimental results to predict complex AHL-receptor interactions occurring in microbial populations in nature.

## Results

### Detecting quorum sensing crosstalk using the plate-assay setup

Our experimental setup was based on a LuxI/LuxR sender-receiver type plate assay [14,20,24,25,27,28] with the addition of an interacting strain. In this setup, the sender strain produces the AHL 3-oxo-C6 HSL [21], which then binds to the receptor protein LuxR and activates gene expression in the senders when there is a sufficient amount of AHLs present. Activated gene expression in the senders results in elevated GFP production, see Fig 1A. The receivers have the capability of producing the LuxR receptor protein, and in the presence of a sufficient amount of AHLs, they will activate gene expression of RFP. The interactors constitutively produce a non-cognate AHL corresponding to the *lasI* or *rhlI* synthase genes. The plasmids encoding these constructs are shown in Figure A in S1 File. Coculture of the receiver and the sender resulted in a threefold increase in fluorescence and the introduction of the interactors produced an equivalent or reduced level of fluorescence, see Figure B in S1 File.

Receiver cells containing a LuxR regulated fluorescent reporter were distributed on a 20 mm diameter LB agar pad containing a sender strain colony in the middle of the plate, see Fig 1B. The sender contains a plasmid expressing the LuxRI circuit, see Figure A in S1 File. The activation times of the gene expression in the receivers were measured with respect to the distance from the sender colony. By adding an interacting strain producing an additional AHL into the lawn of receiver cells, the shift in the activation profile quantifies crosstalk between the interactor strain and the sender/receiver system.

As shown in Fig 1C, in the plate assay receiver cells adjacent to the senders express RFP around 5.25 hours and the receiver cells located at larger distances activated RFP after 6–8 hours. To quantify this propagation of activation within the plate, RFP fluorescent images were used to calculate the time it takes to activate RFP expression in the receivers at multiple distances from the sender colony. Activation of RFP is defined as when at least 10% of the pixels belonging to cells have a pixel intensity greater than a threshold value. Activated cells displayed a clear increase in fluorescence intensity (Figure B in S1 File) and the measured activation times were not sensitive to small changes in the threshold intensity used in image analysis, see Figure C in S1 File.

As mentioned previously [9], there are two main types of AHL crosstalk mechanisms between microbial species as shown in Fig 1D. In the context of crosstalk, the senders will produce the cognate AHL for the receivers while the interactors will produce a non-cognate signal variant, which binds to the LuxR receptor. For excitatory crosstalk, the interacting species are promoting the activation of the receivers while for inhibitory crosstalk, the interacting species are repressing the activation.

**Fig 2:**
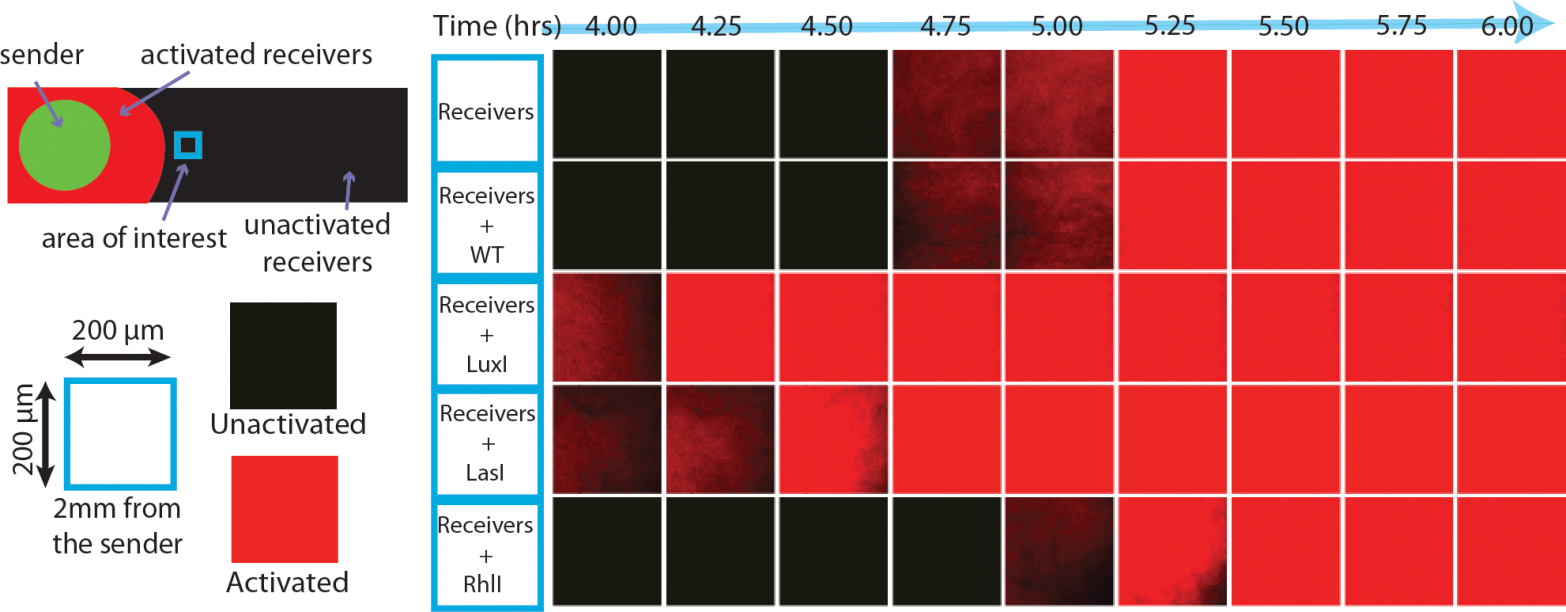
The assay captures a range of crosstalk behaviors. Schematic of the crosstalk assay. A kymograph of fluorescence expression in the receiver cells at a position 2 mm from the sender colony for five different conditions. The squares show fluorescent images taken between 4 and 6 hours. The top two lines demonstrate that the addition of an interactor strain that does not produce any signal, WT, did not change the activation time. The addition of an interactor strain containing a signal synthase gene (bottom three conditions) shifted the time of activation.

The introduction of an interactor species to the sender-receiver setup should shift the activation times of the receivers. As an initial positive control shown in Fig 2, sender cells containing *luxI* were added to the lawn of receivers to verify a decrease in activation time, by 0.75 hr, for an interactor strain producing the cognate signal. In a negative control where the interactor strain is the wild type *E*. *coli* host strain not producing any AHL signal, the activation time is unchanged. This result indicates that the space taken up by interactor cells did not affect the response of the receiver cells (see Figure D in S1 File).

We tested crosstalk from non-cognate signals by introducing an *E*. *coli* strain containing the synthase genes *rhlI* or *lasI*, producing C4-HSL and 3-oxo-C12-HSL as their principle products, respectively [9,23]. We chose these two AHL circuits based on the evidence of both excitatory and inhibitory crosstalk with the LuxI/R system from previous studies [17,20,23,29]. As shown in Fig 2, when the *E*. *coli* LasI strain was introduced as the interactor strain, the receivers activated RFP earlier compared to the no crosstalk control. When the *E*. *coli* RhlI strain was introduced as the interactor strain, the receivers activated RFP later. These initial tests confirmed that both inhibitory and excitatory crosstalk could be observed in our assay. As seen in Figure E in the S1 File, the introduction of these interactor strains does not influence the growth of the sender or receiver strains, supporting the conclusion that the observed effect was due to the crosstalk among the AHLs and receptor.

### Measuring the scaling of crosstalk delay with community composition

**Fig 3:**
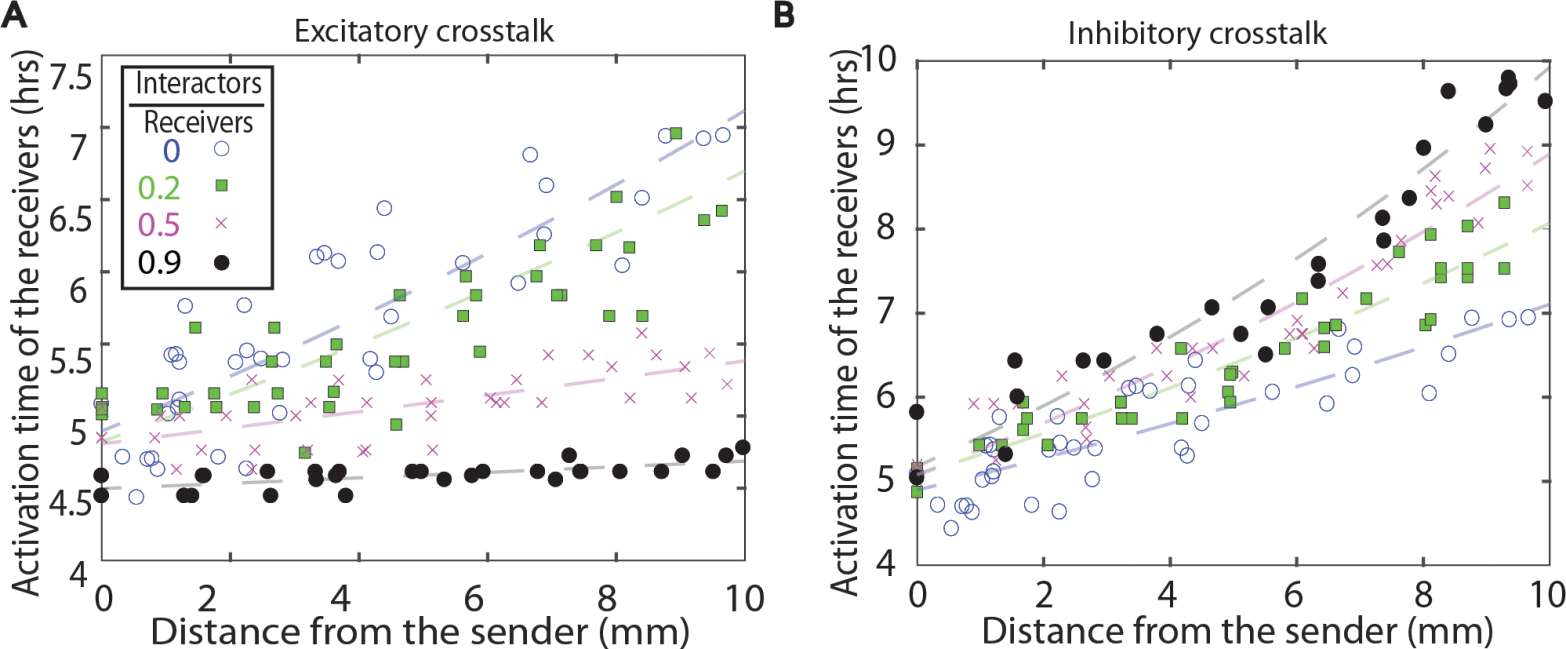
The dependence of activation dynamics on the number of interactor cells. **A.** The addition of the LasI strain as the interactor strain reduces the activation time compared to case of no crosstalk. **B.** The addition of the RhlI strain as the interactor increases activation times compared to the no crosstalk case. In both cases, the shift in activation time was proportional to the amount of interactor strain added, as defined by the ratio of interactor to receiver (see text for details). Experimental data are from three independent measurements. The plots in the background show the trend of the data and are solely meant to guide the eye.

The delay in the activation of the receiver strain as a function of the number of interactor cells was measured by varying the amount of interactor strain loaded onto the plate. The amount of interactor strain added to the plate is captured as the interactor to receiver ratio, which is defined as the ratio of the number of interactors cells loaded on the plate assay to the number of receiver cells loaded on the plate assay. The number of receivers was always kept constant at 10^8^ cells. As shown in Fig 3, the shift in the activation time was proportional to the amount of interacting cells. The activation curves are shown for the cases of excitatory crosstalk (Fig 3A), with LasI as the interactor strain, and inhibitory crosstalk (Fig 3B), with RhlI as the interactor strain. These experiments quantify how the propagation of the activation front depends on the community composition, both in terms of the types of signals produced and the relative amount of each interactor strain in the environment.

### Modeling quorum sensing crosstalk with a reaction-diffusion equation

In this section, we build a mathematical model to explore the correlations of the micro level binding of a signal to a receptor, and the macro level spatiotemporal patterns of gene expression in a system incorporating quorum sensing crosstalk. In previous work done by [21,22,28,30–32], the authors have implemented a logistic growth Eq (1) and a reaction-diffusion model (2) to simulate signal production from growing cells. The logistic growth equation considers the transient behavior of the cell density *n*_*i*_, which is growing at a rate of μ per cell. As the media has a finite amount of resources, the total cell density (senders + receivers + interactors), *n*_*T*_, will approach a saturated cell density of s.

$$\frac{\partial n_{i}}{\partial t}=\mu \;n_{i}(1-\frac{n_{T}}{s}).$$ (1)

Initially the senders produce the AHLs at a basal rate of *ρ*_*b*_ per cell. The sender AHLs, with a concentration of *c*_*s*,_ diffuse away from the cells with a diffusion coefficient of *D*_*c*_ and are degraded by the media at a rate of *d*_*a*_. *ρ* accounts for an increase in AHL production in the presence of signal. For the senders, the activity (A) defines how this activated rate of signal production, due to changes in production of the synthase protein, depends on the concentrations of multiple AHLs.

$$\frac{\partial c_{s}}{\partial t}=D_{c}\nabla^{2}c_{s}+n_{s}(\rho \;A+\rho_{b})-d_{a}c_{s}.$$ (2)

In the presence of an interacting AHL, the transcriptional activity will be modulated due to the binding of AHLs to the LuxR receptors. Each signal has a variable influence on the activity, both in the ability to bind to a receptor and the downstream influence of such binding on the expression of quorum sensing controlled genes. The ability of an AHL to bind to the LuxR receptor depends on the binding energy and the local concentration of each AHL. In simulations, we considered the probability of an AHL to bind to a receptor and introduced a weight to account for the downstream influence of each AHL variant on gene expression. Therefore, the activity takes the form of,

$$A=g\;(\sum_{i=0}^{j}P(c_{i})w_{i}),$$ (3)

where, g is the number of receptors per cell, *i* is the index to describe the type of AHL, *c*_*i*_ is the concentration of the i^th^ AHL, *P*(*c*_*i*_) is the probability of an AHL binding to the receptor, *w*_*i*_ is the weight parameter and j is the total number of interacting AHLs. The probability of binding accounts for differences in the binding affinity of each signal variant to the receptor, as well as the competition for multiple signals to bind to the same receptor. The number of receptors (g) changes from a basal level of 100 to 600, as gene expression levels increase due to signal accumulation. To model this smooth transition we used a Hill’s function, see Table A in S1 File. It is only physical to have zero or positive levels of transcriptional activity, therefore the weights should also be greater than or equal to zero.

The weight parameter (*w*_*i*_) relates the number of AHL-receptor complexes to the extent of gene regulation, with large positive weights indicating that complexes formed by that AHL lead to strong upregulation of quorum sensing regulated genes while weights close to zero lead to inhibition of these genes. The weight is determined by the affinity of the bound receptor for the promoter region of quorum sensing regulated genes, the efficiencies of transcription and translation of quorum sensing regulated genes, and the rate of dissociation for the AHL-receptor complex. A deterministic Boltzmann weight approach was applied to calculate the receptor binding probabilities from AHL concentrations and receptor binding energies [33], see the mathematical model in Text A in the S1 File and Figures F-H in the S1 File. Parameter values given in Table A in the S1 File were measured in control experiments or obtained from previous experimental studies [19,31,34–36].

Since the interactor strain did not produce any receptors, signal production was constitutive and did not incorporate positive feedback from AHL level. The activity (A) for the interactors was zero and signal production occurred at a basal level,

$$\frac{\partial c_{int}}{\partial t}=D_{c}\nabla^{2}c_{int}+n_{int}\;\rho -d_{a}c_{int}.$$ (4)

The constitutive production rate was assumed to have the same value as the maximum production rate of the senders. These equations were solved using the finite difference method. The model predictions were obtained considering the transient behavior of the AHL concentrations. In simulations, we considered two concentric circles; the inner circle has a radius of 1 mm while the outer circle has a radius of 10 mm. The cell densities are governed by Eq (1), the dynamics of the signals of the senders are governed by Eqs (2) and (3), and the interactors by Eq (4). The initial conditions for simulations were chosen to mirror experimental conditions. As in experiments, initially 10^7^ sender cells were added to the inner circle. The inner circle was assumed to have an initial AHL concentration of 70 nM. The outer circle has a variable mixture of receivers and interactors distributed evenly in space. In all cases, there were 10^8^ receivers cells. The initial concentration of the interactor AHL in the outer circle was 70 nM [31]. In simulations, the amount of interactor strain was adjusted, as specified by the interactor to receiver ratio.

Based on the diffusive gradients of signals created by the senders and the interacting species, the activity of the receivers was calculated using,

$$A_{R}=g(P(c_{S})w_{s}+P(c_{int})w_{int}),$$ (5)

where, *P*(*c*_*S*_) is the probability that the AHLs from the sender will bind to the receptor, *P*(*c*_*int*_) is the probability that the AHLs from the interactor will bind to the receptor, *w*_*s*_ is the weight associated with the sender AHL and *w*_*int*_ is the weight associated with the interactor AHL. The activity of the receivers modulates the production of the fluorescent gene reporter (RFP), as the reporter gene is transcribed by a promoter regulated by signal bound receptor.

The level of activity of the receivers acts as an indicator of changes in gene expression resulting from the crosstalk. Therefore, we define a threshold activity level for the activation of gene expression and used Eq (5) to track whether the activity level of the receivers exceeded this threshold. In simulations, the threshold activity was taken to be half of the maximum activity level when there is no crosstalk.

### Robustness of signal transduction to crosstalk

**Fig 4:**
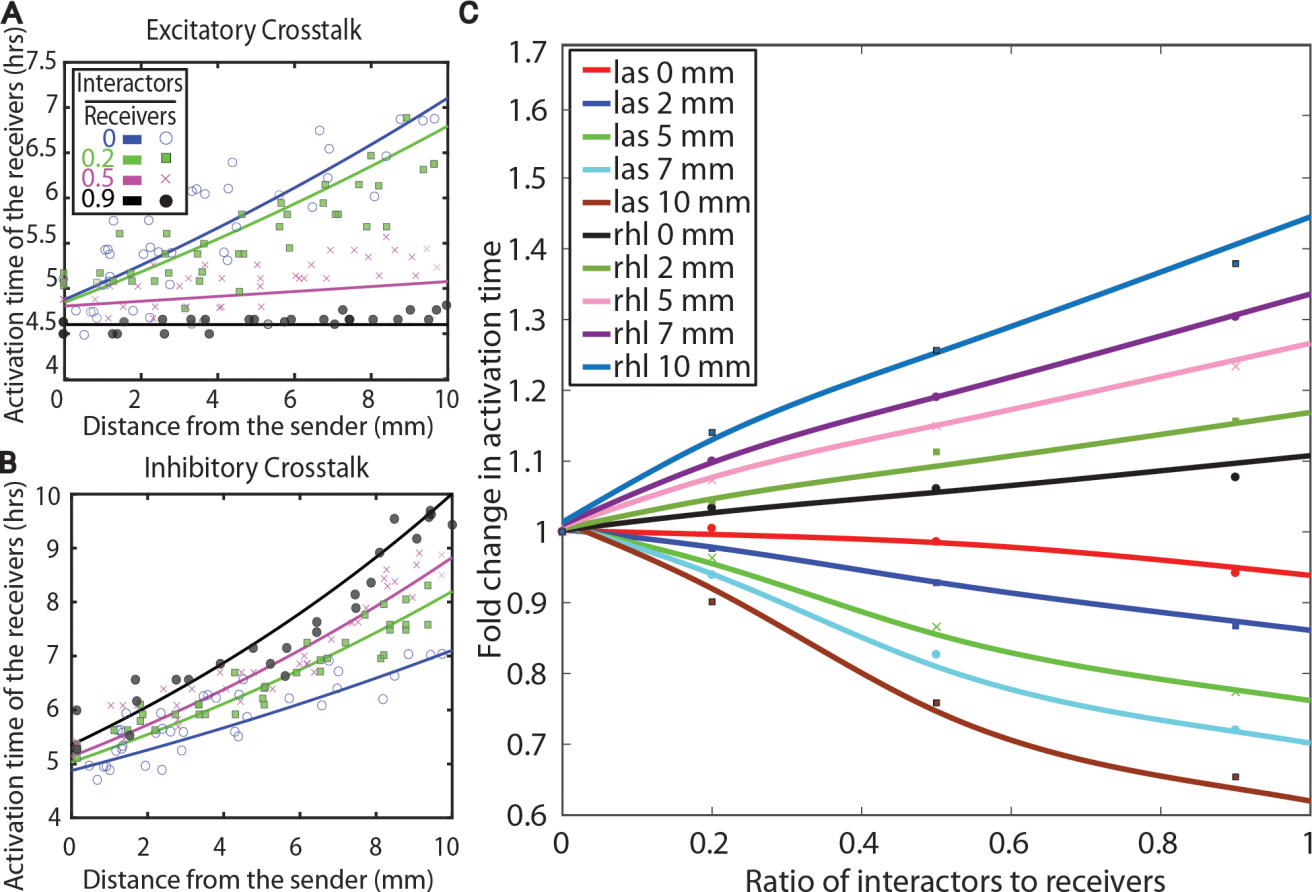
Robustness of the activation of gene expression to crosstalk. **A,B.** Theoretical predictions for the response of the receiver cells in the presence of excitatory crosstalk or inhibitory crosstalk. The experimental data from Fig 3 are shown for comparison. **C.** Comparisons between the experimental measurements of the activation of gene expression in the plate assay to predictions made using the reaction diffusion model. Lines show the predicted change in the activation time at multiple distances from the sender colony as a function of the amount of interactor strain added to the plate. Predictions were made using the experimentally calculated crosstalk weights for the RhlI and LasI interactor strains. Data points show experimental measurements at selected distances from Fig 3.

To obtain signal weights of the sender AHL and the two interacting AHLs, simulation results were fit using the experimental data from Fig 3. The weight parameter (w_1_) for the signal 3-oxo-C6-HSL binding to the LuxR receptor was fit using the experimental data for no crosstalk. Non-linear least square fitting method was used for this purpose, see Figure Ia in S1 File. Additional weight terms are needed to account for each interacting AHL. To identify the weight parameters of the interacting AHLs, experimental plots shown in Fig 3 were fit for the case of 0.9 ratio of interactor to receiver using the non-linear least square fitting (Figure I in S1 File). Calculated weights for signals produced by LasI and RhlI interactor strains are shown in Table 1. Using these weights, activation curves for the ratio of interactor to receiver of 0.2 and 0.5 were simulated, as shown in Fig 4A and 4B, revealing a scaling similar to experimental data shown in Fig 3.

**Table 1:** The weight parameters derived from the comparison of the model simulations and the experimental results.

Synthase	Type of AHL	Weight
LuxI	3-oxo-C6-HSL	*w*_*Lux*_ = 0.701 ± 0.016
LasI	3-oxo-C12 HSL	*w*_*Las*_ = 0.400 ± 0.013
RhlI	C4-HSL	*w*_*Rhl*_ = 0.002 ± 0.001

These values represent the regulatory response of each type of AHL bound to the LuxR receptor protein. The error represents the standard deviation for the weight values.

The validated model of crosstalk has enabled the exploration of the robustness of signal propagation. Signal propagation in the presence of variable levels of crosstalk was simulated for both the LasI and RhlI interactor strains. Fig 4C shows the predicted delay in the activation of the receiver strain at distances of 0, 2, 5, 7 and 10 mm for ratio of interactor to receiver values between 0 and 1. Data points show experimental measurements of the activation of the RFP response at those distances and crosstalk levels, revealing a good agreement with model predictions.

Using the model, we predicted the sensitivity of signaling dynamics to changes in model parameters, including cell growth rate (Figure J in S1 File), signal production rate (Figure K in S1 File), diffusion coefficient (Figure L in S1 File), and the signal degradation rate (Figure M in S1 File). Activation times strongly depended on the diffusion coefficient, signal production rate, and signal degradation rate, as together these parameters set the concentration profile of the signal. Growth rate did not affect the activation times, likely because cells were loaded onto the plate at a density near saturation, so few divisions took place during the experiment. Additionally, we observe that the crosstalk is highly correlated to the binding energy and the weight parameter, see Figure N and Figure O in S1 File. The activity of the receiver strain for variable concentrations of the signals made by the sender strain and the interactor strain is also plotted in Figure P in the S1 File. The model predicts that the activity of the senders are unaffected by signal exchange with the interactors, see Figure Q in the S1 File.

### Measuring the crosstalk potential of wild isolates

**Fig 5:**
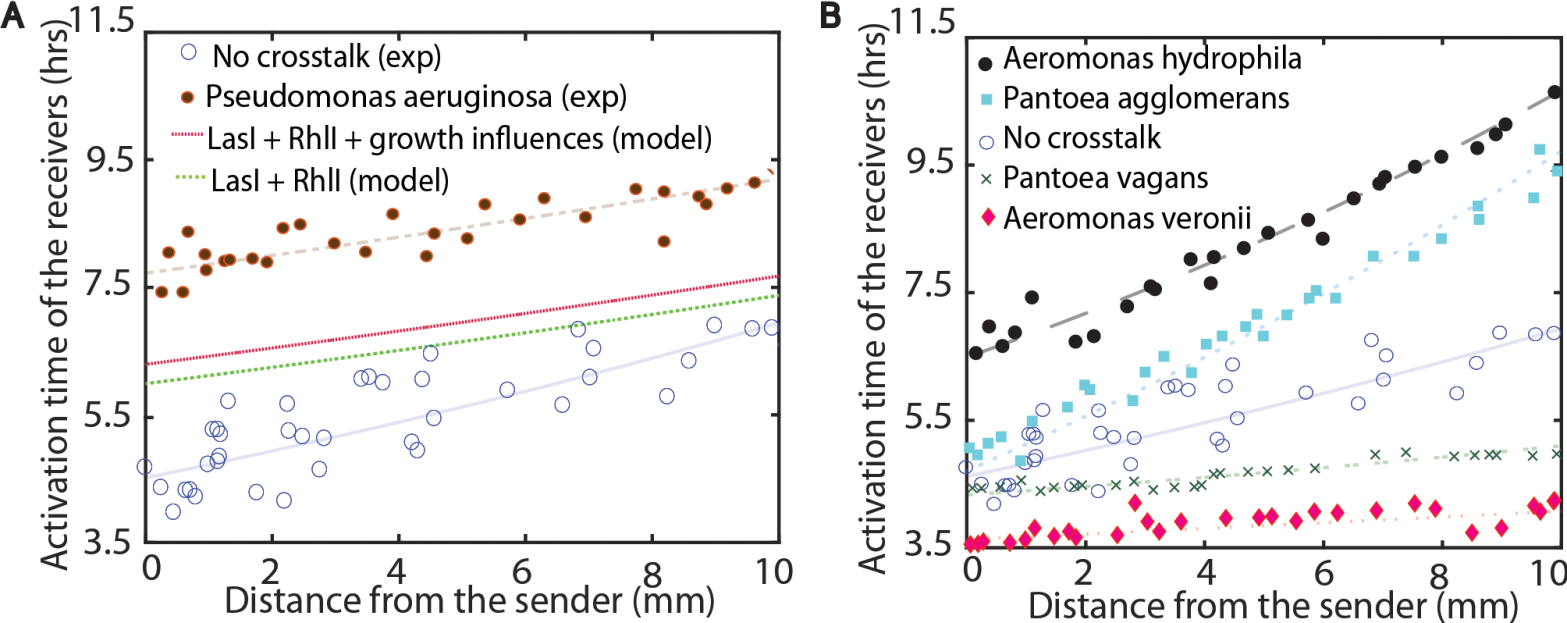
Testing the model and experiments with natural isolates. **A.** The comparison of simulation results to the experimental results with *Pseudomonas aeruginosa* as the interactor strain. Data from the LasI and RhlI interactor strains were used to predict the combined influence of the LasI and RhlI quorum sensing circuits in *P*. *aeruginosa*. The LasI + RhlI + growth influences line adds the experimentally measured reduction in the growth rate of *E*. *coli* in the presence of *P*. *aeruginosa* to the model, see Figures R-T in S1 File. **B.** The plate assay was used to measure the interference potential of four wild bacterial isolates at 0.9 ratio of interactor to receiver. Lines, shown to guide the eye, are exponential fits to the data.

The assay also enables measurements of crosstalk with wild isolates. As an initial test, crosstalk with wild type *Pseudomonas aeruginosa* was measured, as shown in Fig 5A. The presence of *Pseudomonas aeruginosa* at 0.9 ratio of interactor to receiver delays activation by several hours. Because the *las* and *rhl* genes are derived from *Pseudomonas aeruginosa*, the weight parameters extracted from the *E*. *coli* interactor strains were used to predict the expected delay in activation as a result of crosstalk with these two systems. We simulated interactions with a hypothetical interactor strain containing both the *las* and *rhl*. Here the activity for the receivers will be,

$$A_{R}=g(P(c_{Lux})w_{Lux}+P(c_{Las})w_{Las}+P(c_{Rhl})w_{Rhl}).$$ (6)

In Fig 5A, we observed that the trend of the simulated activation curve is similar to the experimental results, showing delayed activation and a shallower activation curve across the plate. The predicted delay was shorter than the experimentally measured delay by approximately 2 hours. In the S1 File, additional simulations are performed to determine if a delay in growth of the sender strain or the influence of the sender strain AHL on AHL production in *P*. *aeruginosa* might contribute to the additional delay in activation. A reduction in the sender strain growth rate, when cocultured with *P*. *aeruginosa*, was confirmed in growth measurements, see Figure R in S1 File. Fig 5A shows the prediction of the model that incorporates growth influences. Although QS activation was delayed, growth interactions alone were not sufficient to reproduce the 2 hour delay in activation, see Text B, Figure S and Figure T in S1 File.

Next, the crosstalk potential of four additional wild isolates was tested. The four were added to the plate assay at 0.9 ratio of interactor to receiver. 16S rRNA sequencing identified the wild type species as *Aeromonas hydrophila*, *Aeromoans veronii*, *Pantoea agglomerans*, and *Pantoea vagans*. The ability of these strains to produce AHL has been reported previously [37–42]. In Fig 5B, we observed that when A. *veronii* or P. *vagans* were added to the lawn of receiver cells, the activation of the RFP in the receivers was earlier as compared to no interactor strain. A. *hydrophila* and P. *agglomerans* both delayed activation. The extent of crosstalk was different for each species, suggesting that the activation of genetic expression in diverse communities is likely influenced by crosstalk of variable strength from multiple species.

## Discussion

Our results give new insights into signaling within mixed communities of bacteria. Adapting an approach used in previous studies [24,25,28,43–45], we created a sender-receiver type plate assay to quantify the activation of gene expression due to AHL-mediated signaling in the presence of multiple signal producing strains. The assay measured the robustness of specific signaling networks to interference by a strain producing a non-cognate signaling molecule. When comparing the spatial reaction-diffusion based assay to well-mixed systems, we found that the spatial assay is able to differentiate an interactor strain that produces a non-cognate signal from a strain that produces a signal destroying enzyme, see Figure U in S1 File. Although crosstalk between quorum sensing networks has been previously reported [9,11,12,23,46,47], our titration of the interacting strain revealed the sensitivity of signal-mediated gene expression in a spatially distributed network to interference. As shown in Fig 4C, at distances 2 mm or less, the fold change is less than 10% even for crosstalk ratios of 1. At distances of 10 mm, the activation time has changed by approximately 10% at only 20% ratio of interactor to receiver. These numbers suggest that quorum sensing based genetic activity is largely robust to interference and that any abundant species should activate its quorum sensing network in a typical system. Our results are specific to LasI and RhlI interference with LuxRI QS system, and it is yet unclear if some AHL systems would have evolved differing levels of robustness or sensitivity to particular non-cognate signals.

The model demonstrates the robustness of the AHL network to interference is in part due to differences in the binding energies of cognate and non-cognate signals. Figure N in the S1 File shows that as the binding energy of the non-cognate signal weakens, crosstalk from the non-cognate signal has little effect on gene regulation. The ratio of interactors to receiving cells also influences robustness. As shown in Figure V in the S1 File, when interactor cells greatly outnumber the receiver cells, robustness is lost and the expression of LuxR/I regulated genes is delayed by several hours. A third factor affecting robustness is the interaction weight for the non-cognate signal, as shown in Figure O in the S1 File. Future work should further characterize the range of interaction weights present in real systems. A better understanding of the robustness of signal exchange in mixed populations would be beneficial to the implementation of quorum sensing gene circuits in synthetic microbial communities [2,15,18–20,44].

Our experimental measurements aided in the development of a detailed model to predict AHL-based signaling dynamics in mixed populations. The model accounts for crosstalk between strains using a single parameter called the weight that we calculate from experiments for a given receptor for each combination of signals. This weight accounts for the downstream regulatory consequences of a receptor binding to the AHLs, and would be related to fundamental processes such as receptor dimerization, interactions between the receptor, DNA, and RNA polymerase, and the transcription and translation of the AHL-regulated genes. We found that a model using a single weight value was in good agreement with experimental activation dynamics covering over 1 cm of space with variable amounts of interference. This close agreement between the model and experiments suggests that the model can be implemented to examine quorum sensing crosstalk in more complex and realistic contexts, such as in the presence of more than two strains, when cells are heterogeneously distributed in space, or even when transport dynamics are spatially dependent [21,25,30]. Since the experiments in these contexts would be challenging, our assay and model provide a straightforward path towards predicting signaling dynamics in complex conditions. In addition to predicting the dynamics in complex conditions, as mentioned above, the model has enabled an exploration of how robustness to interference might emerge by adjusting the parameters that regulate the response to signal exchange. Robustness can be achieved if the receptor has evolved to bind the non-cognate signal much more weakly than the cognate signal. The difference in the receptor binding energies between the non-cognate and cognate signals needed for robustness is influenced by the number of interactor cells and the influence of the non-cognate signal on gene expression, as captured in the weight term. Some receptors may have evolved a sufficient amount of binding discrepancy based on interactor strains and non-cognate signals typically encountered.

Our analysis of signal interference with wild species revealed a wide variety of crosstalk patterns within natural populations. We found both excitatory and inhibitory crosstalk within our isolates and a variable extent of crosstalk with the luxRI quorum sensing system. Previous measurements have also shown that non-cognate AHLs can interact with receptors such as LuxR to varying degrees [9,23]. Because we use the wild isolate directly instead of purified signal, cell free supernatant, or synthetic producer strains, we capture both direct and indirect signaling interactions with the interacting strain. Examples of indirect interactions include modulation of growth rate and gene regulatory pathways, and the evolving spatial distributions of the interactor. We attempted in the case of the interaction with *Pseudomonas aeruginosa* to specify the source of these indirect signaling interactions by independently accounting for the influence of each AHL signal produced by the interactor strain and growth influences of the interactor strain on the receiver cells. Although the model predicted an increased delay in activation due to growth effects, as shown in Fig 5A, there are still additional currently unknown interactions that further delay activation. We speculate that the QscR receptor residing in *Pseudomonas aeruginosa* might be absorbing the sender AHLs and contributing to this delay [48], although other non-AHL based regulatory interactions between species likely contribute to signaling dynamics. Future efforts should attempt to disentangle the direct and indirect interactions that influence signal transduction to improve our ability to predict signaling dynamics in real populations. In addition, for some ecological niches, growth dynamics and cell movement can affect the AHL gradients in unexpected ways and these factors should be incorporated to any future work to understand signaling dynamics in complex environments [49,50]. Using the assay to broadly sample interactions between known AHL signal-receptor system, such as luxRI, and wild signal producers should yield new insights into patterns of crosstalk within real environments and their consequences in ecosystem level regulation of quorum sensing.

## Materials and methods

### Bacterial strains and plasmids

In Table 2, we have represented the details of the bacterial strains used in this study. The host strain used for the sender, receiver and interactors are *Escherichia coli* NEB 5-alpha. The major QS signals are 3-oxo-C6 HSL for the sender strain, 3-oxo-C12 HSL for the LasI interactor strain, and C4-HSL for the RhlI interactor strain, see Figure A in S1 File for further details. The plasmids were either obtained from Addgene [21] or constructed using Gibson assembly (New England Biolabs).

**Table 2:** The bacterial strains used in this study.

Species or strain	Plasmid	Obtained from
*Escherichia coli sender*	ptD103LuxI sfGFP	[21]
*E*. *coli receiver*	ptD103LuxR RFP	[35]
*E*. *coli LasI interactor*	pZE2501Las	this study
*E*. *coli RhlI interactor*	pZE2501Rhl	this study
*Pseudomonas aeruginosa*		[51]
*Aeromonas hydrophila*		[52]
*Aeromonas veronii*		[52]
*Pantoea agglomerans*		this study
*Pantoea vagans*		this study

### Culturing conditions

The bacterial strains were inoculated from frozen stocks in a 12 ml Falcon tube with 5 mL of LB broth with appropriate antibiotics. The inoculum was grown in a shaker at 220 RPM at 37°C for 16 hours. Cells were resuspended in fresh media to remove signal in the supernatant. Late log phase cultures were used such that quorum sensing of the sender strain was activated before measurement in the plate assay.

### Plate assay

The plate assay was setup as described in Silva et al [35]. The interactor strain was mixed with 100 μl of the receivers in a 1.5 mL centrifuge tube and spread onto the top of 2.5% LB agar plates using sterile 4 mm glass beads. Figure W in the S1 File shows that the spatial distribution of cells remained mixed during the assay.

### Microscopy measurements

A Nikon eclipse TI fluorescent microscope was used for image acquisition. Experiments were done at 37°C using a temperature controlled chamber. Samples were imaged at a magnification of 20x. To record the RFP activation in the receiver cells, RFP images were taken every 15 minutes for 16 hours at 30 different distances from the sender colony. Activation times were calculated at each position. Exposure times were 1s for RFP and 500 ms for GFP. No significant photobleaching was observed.

Each image taken was saved in.tiff format and analyzed using a custom Matlab code. A low threshold was applied to the RFP images to identify the location of the receiver cells within each image. An upper threshold was used to identify the receivers that had activated RFP. For each time point and position, the fraction of cellular pixels above the RFP activation threshold was calculated. If the fraction of activated pixels exceeded 10%, that position was included as part of the activated region, see Figure C in S1 File.

### Growth measurements

To obtain growth curves, overnight cultures were diluted 1 to 1000 in LB media and selective plating was performed to measure cell density over time. To obtain growth curves from mixtures of strains, each strain had a unique resistance marker and was plated on the appropriate selection plate.

### Plate-reader measurements

Tecan Infinite m200 Pro plate reader was used to measure growth rates and fluorescence activation in well-mixed conditions. Cells were grown to late log phase, diluted 1000 fold in pure LB media, and cultured for an additional 3 hours. After three hours of growth, 200 μl of these early log-phase cells were loaded into a flat bottom 96-well plate. The plate was inserted into the plate reader set to 37°C and the optical density and fluorescent intensity were measured every 15 minutes for 16 hours. Optical density measurements were carried out at a wavelength of 600 nm. For GFP measurements, a wavelength of 485 nm was used for excitation and a wavelength of 515 nm was used for emission. For RFP fluorescence measurements, a wavelength of 590 nm was used for excitation and a wavelength of 650 nm was used for emission.

## Supporting information

S1 File Contains all the supporting figures, texts and tables for the manuscript. (DOCX)
