# Supplementary material for: Quantifying the strength of quorum sensing crosstalk within microbial communities
Source: PLoS Comput Biol. 2017 Oct 19;13(10):e1005809. doi: 10.1371/journal.pcbi.1005809 (PMC5663516; doi:10.1371/journal.pcbi.1005809)
Supplement: S1 File — (DOCX) [file pcbi.1005809.s001.docx]

**S1 File**

**Quantifying the strength of quorum sensing crosstalk within microbial communities**

**Kalinga Pavan T Silva, Prithiviraj Chellamuthu, and James Q. Boedicker**

**Table of Contents**

**Figure A** Plasmid maps.

**Figure B** Plate reader data for detecting the fluorescent changes in the receivers.

**Figure C** Sensitivity of the results to the activation threshold value.

**Figure D** Control experiments.

**Figure E** Growth rates of the strains and mixtures.

**Text A** Calculating the probabilities of each AHL binding to the LuxR receptor using Boltzmann weights.

**Figure F** A statistical mechanical model of two different AHL signals binding to a signal receptor.

**Figure G** The energy and multiplicity of each unbound and bound state.

**Figure H** The Boltzmann weights for each state.

**Table A** The parameters used in the simulations.

**Figure I** Fitting the experimental data to obtain interaction weights.

**Figure J** Testing the effects of the growth rates of the interactors on crosstalk.

**Figure K** Testing the effects of the production rates of the interacting AHLs on crosstalk.

**Figure L** Testing the effects of the diffusion coefficient of the AHLs of interactors on crosstalk.

**Figure M** Testing the effects of the degradation coefficient of the interacting AHLs on crosstalk.

**Figure N** The influence of the non-cognate AHL binding energy on crosstalk.

**Figure O** The influence of the weights of the AHLs on crosstalk.

**Figure P** Simulated activity profiles of the receivers under well-mixed conditions in the presence of signals coming from both a sender strain and an interactor strain.

**Figure Q** The influence of feedback between interactors and senders on activation of the receivers.

**Figure R** The effect on the growth of the senders and receivers due to *P. aeruginosa.*

**Text B** Testing the model for effects of the growth interactions and AHL internalization.

**Figure S** The impact on crosstalk with non-quorum sensing interactions.

**Figure T** Simulating the impact of *Pseudomonas aeruginosa* growth influences on quorum sensing activation in the receiver strain.

**Figure U** Observing crosstalk and signal degradation in a well-mixed setup.

**Figure V** Robustness of the network to interference when there is a large excess of inhibitory interactors.

**Figure W** Spatial distribution of cells after 16 h.

**References.**


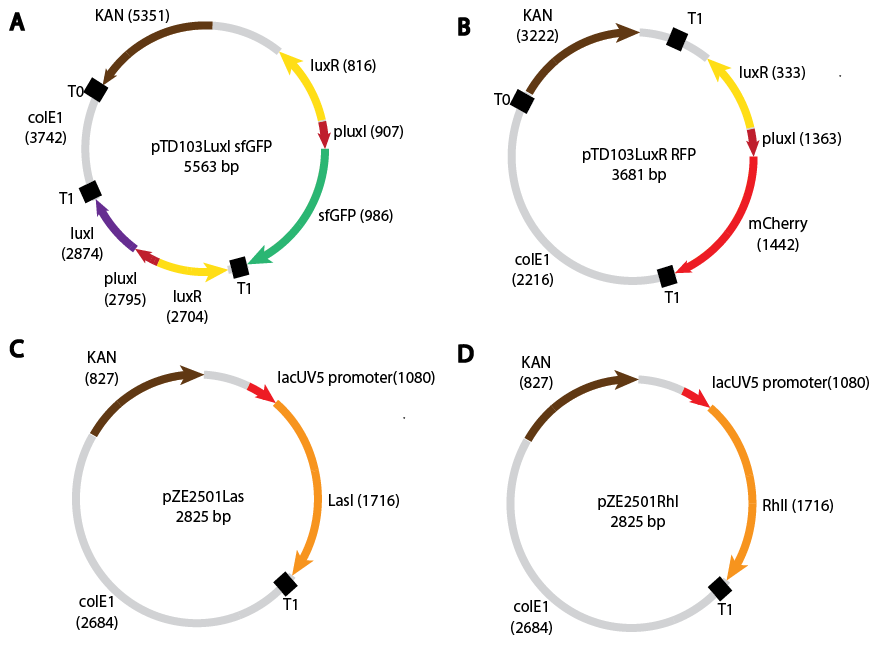


**Figure A. Plasmid maps.** Maps of the plasmids used to construct the **A.** sender, **B.** receiver, and **(C, D)** interactor strains. Sender strain plasmids are from [1]. The quorum sensing promoter *pluxI* drives genetic expression in both the sender and receiver while expression is constitutive in the interactors by the lacUV5 promoter.


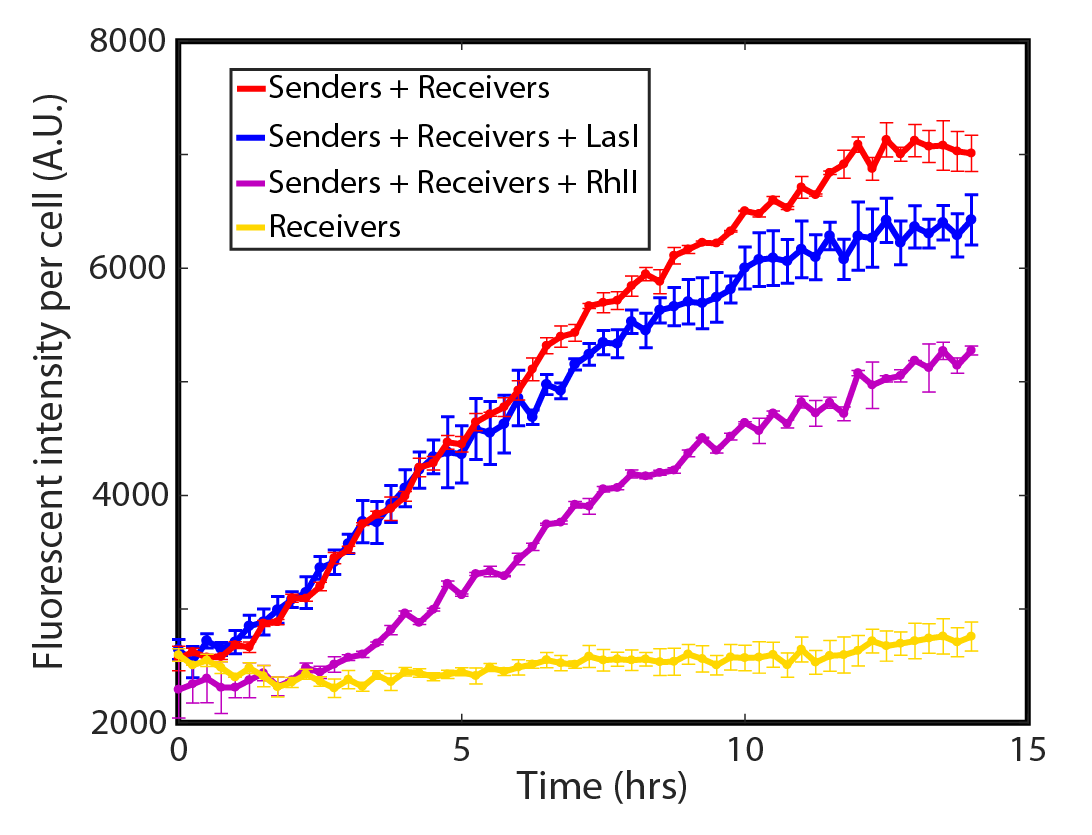


**Figure B. Plate reader data for detecting the fluorescent changes in the receivers.** We have senders (50 µl) + receivers (100 µl) in red, senders (50 µl) + LasI (50 µl) + receivers (100 µl) in blue, senders (50 µl) + RhlI (50 µl) + receivers (100 µl) in magenta and the receivers (100 µl) in yellow. The fluorescent intensity per cell is calculated in these multistrain mixtures as in [2]. The errorbars represents the standard deviation from three sets of replicates.


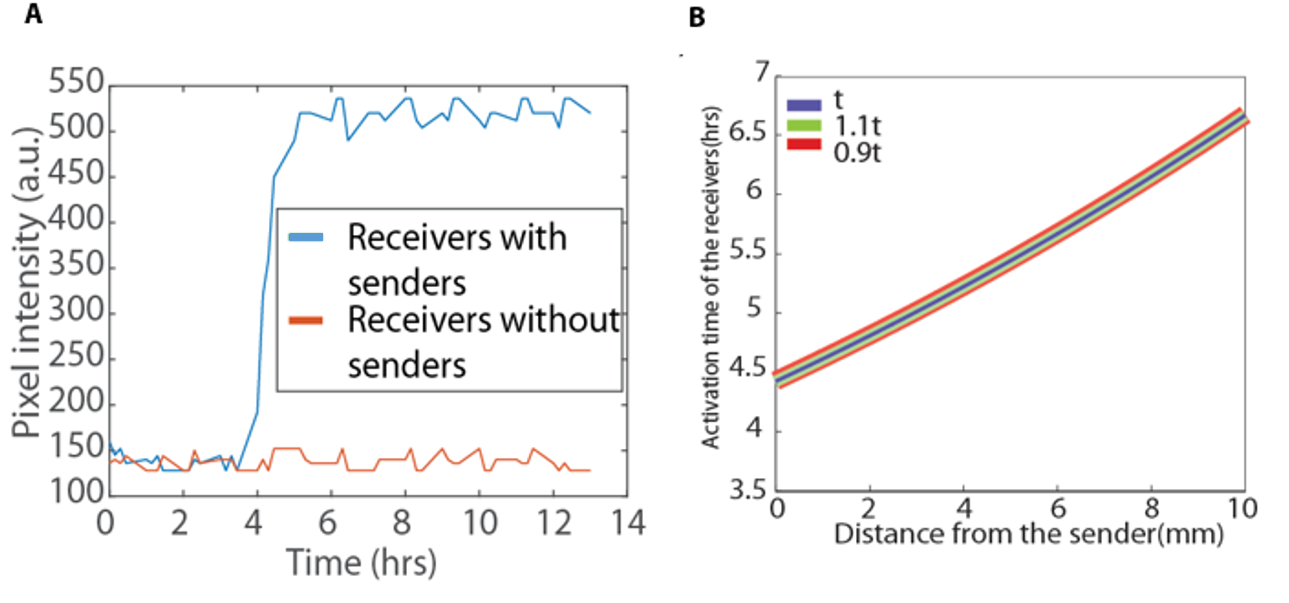
 **Figure C. Sensitivity of results to the activation threshold value. A.** The pixel intensity of the receivers over time at a distance 2 mm from the sender and in a negative control of receivers without added sender. **B.** Lines show exponential fits through the experimental data when reanalyzed using a threshold value (*t*) that was raised or lowered by 10%.


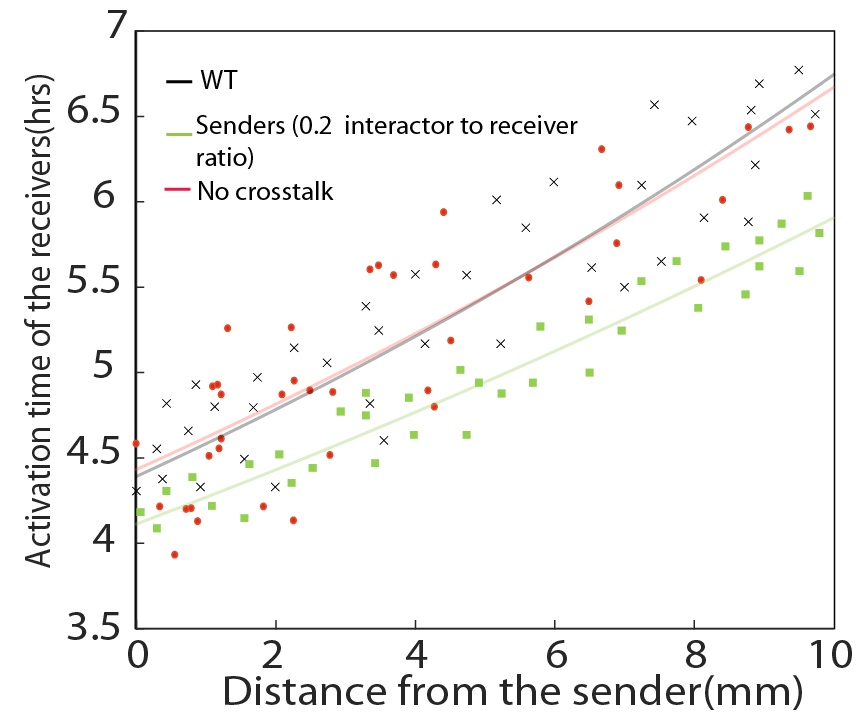


**Figure D. Control experiments.** Activation time vs Distance plots of the no crosstalk case (red), the wild type (WT) acting as the interactor strain at 0.9 ratio of interactors to receivers (black), and additional senders acting as the interactors at 0.2 ratio of interactor to receiver (green). The lines show the trend of the experimental data obtained from three trials.


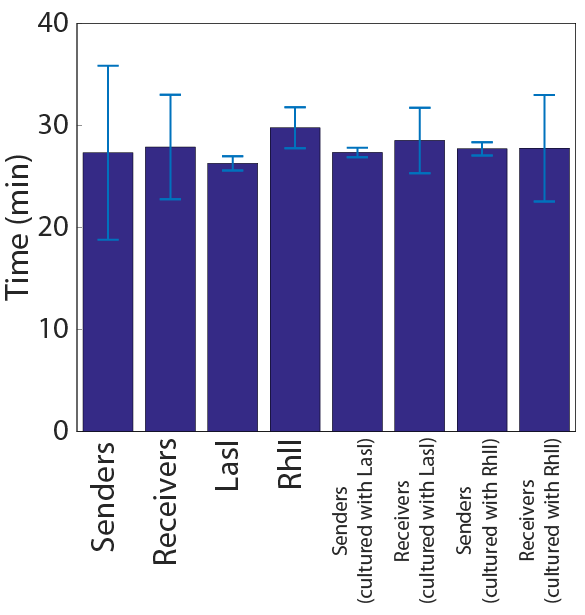


**Figure E. Growth times of the strains and mixtures.** The doubling time of the senders (27.3 ± 8.5 min), receivers (27.9 ± 5.1 min), LasI (26.3 ± 0.7 min), RhlI (29.8 ± 2.0 min), senders cultured with LasI (27.3 ± 0.5 min) , receivers cultured with LasI (28.5 ± 3.2 min) , senders cultured with RhlI (27.7 ± 0.6 min) and receivers cultured with RhlI (27.8 ± 5.1 min). The errorbars represent the standard deviation from three sets of replicates.

**Text A. Calculating the probabilities of each AHL binding to the LuxR receptor using Boltzmann weights**

For simplicity, we will consider a situation where there are two different types of AHLs binding to a single receptor, see Figure F in S1 File.

The energy of the unbound state depends on the unbound AHLs floating in the total solution. We assume that a single AHL has an energy of $\varepsilon_{sol}$ in solution. Therefore, the energy of the unbound state is the total number of unbound AHLs multiplied by $\varepsilon_{sol}$. The AHL binds to a receptor with a binding energy of $\varepsilon_{b}$. The bound AHL will change the number of unbound AHLs by one. Figure F in S1 File depicts the possible states of two different types of AHLs binding to a single receptor.


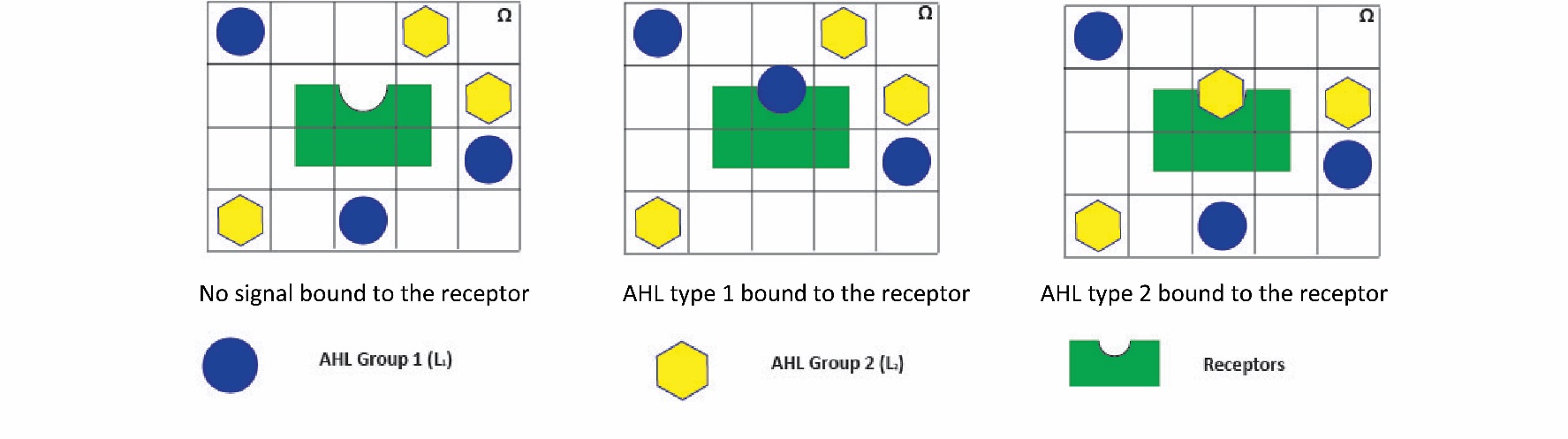


**Figure F. A statistical mechanical model of two different AHL signals binding to a signal receptor.**

Using the states discussed in Figure F in S1 File, we find the energy and multiplicity of each signal being bound to a receptor by implementing the Boltzmann statistics as shown in Figure G in S1 File.

Energy Multiplicity
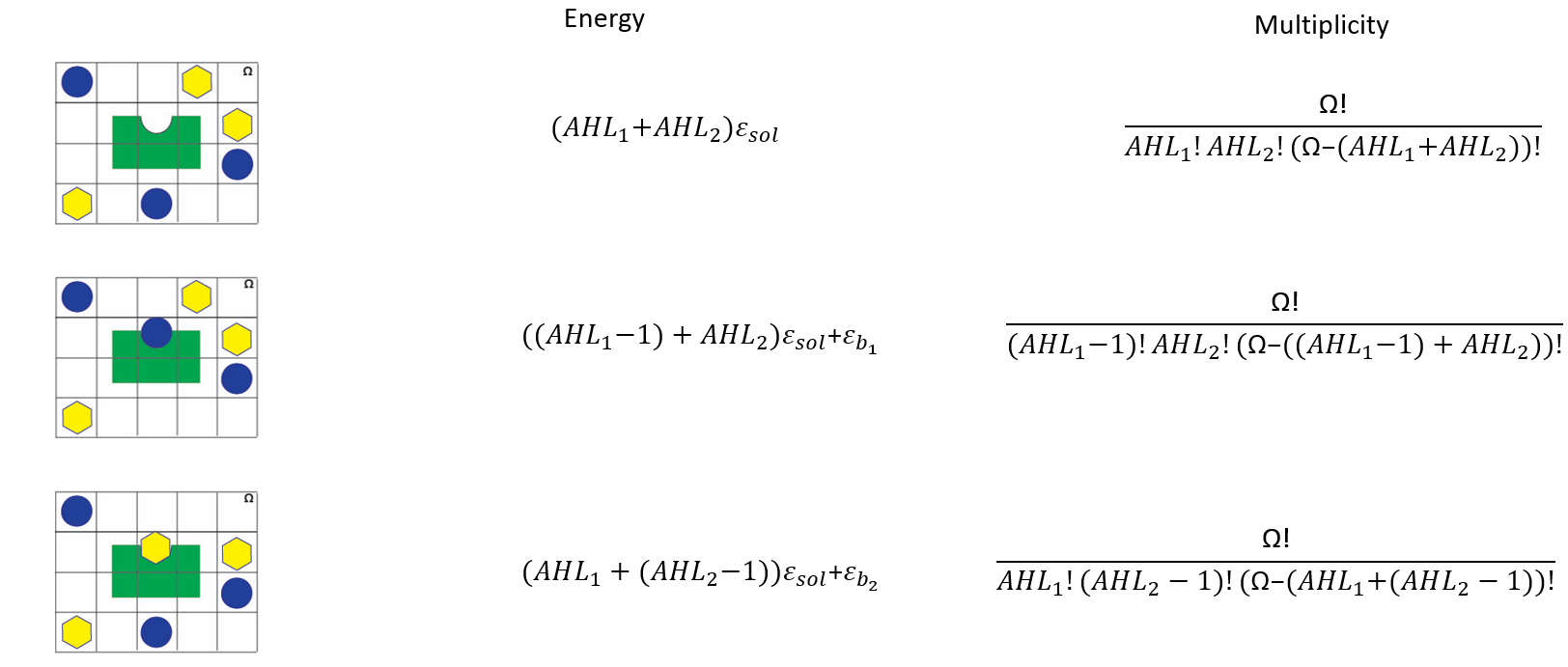


**Figure G. The energy and multiplicity of each unbound and bound state.**

Here,

$\varepsilon_{sol}$ - Energy for an AHL in solution

$\varepsilon_{b_{1}}$ - Binding energy of AHL type 1

$\varepsilon_{b_{2}}$ - Binding energy of AHL type 2

*Ω* - Number of available sites

$\mathrm{AHL}_{1}$ - Number of AHL type1

$\mathrm{AHL}_{2}$ - Number of AHL type 2

As discussed in [3], to calculate the probability of a particular state occurring, we need to consider the Boltzmann weights (these are different from the weight terms in the Activity) of each particular scenario separately, see Figure H in S1 File.

Multiplicity Boltzmann weights


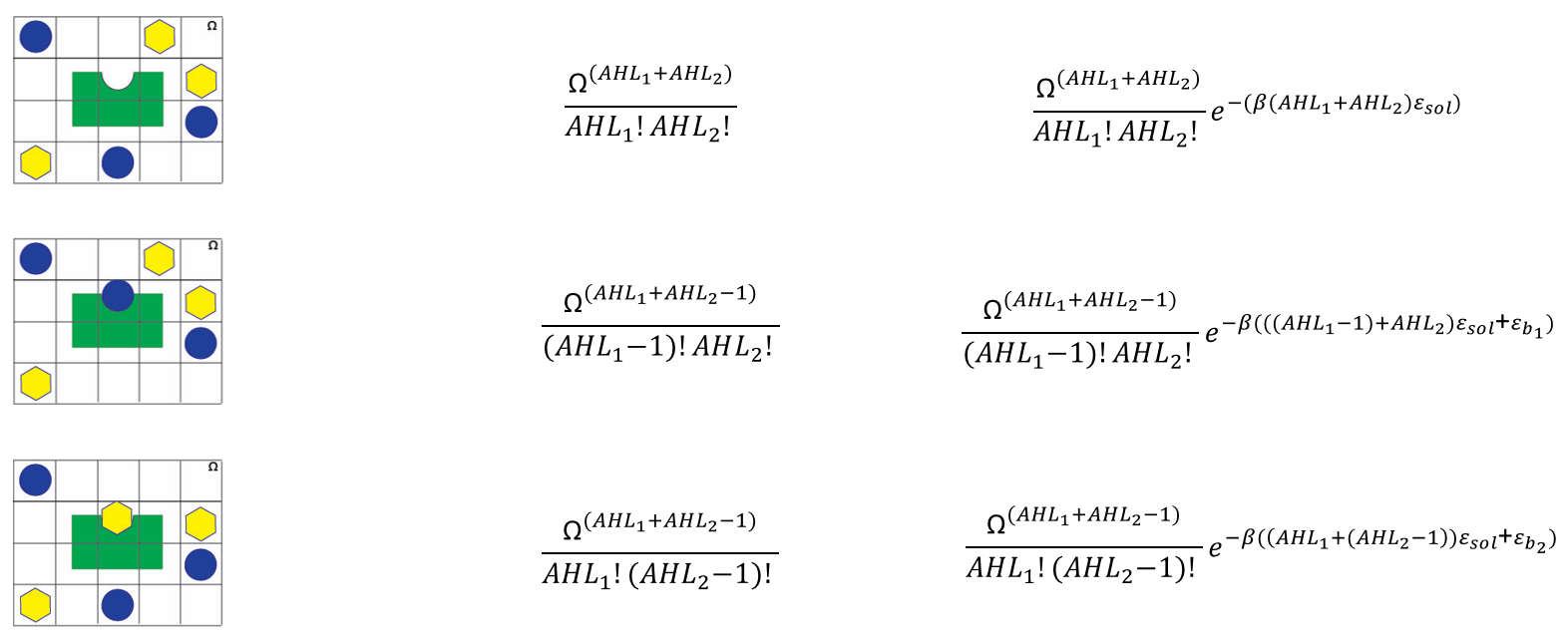


**Figure H. The Boltzmann weights for each state**. Here, $\beta= \frac{1}{k_{B}T}$, where k_B_ is the Boltzmann constant and T is the temperature.

In Figure H in S1 File, we simplified the multiplicity by considering that $\text{Ω}$ >>AHL; $\frac{\text{Ω}\text{!}}{(\text{Ω}\text{-}\text{AHL}))!}$ ~ $\text{Ω}^{AHL}$. This is a valid assumption to make considering the fact that the number of available states will be much higher than the number of AHLs present in the system. Now that we have the Boltzmann weights of each state, calculating the probabilities is straightforward.

The probability of AHL type 1 binding to the receptor is then,

P(${AHL}_{1}$)$=\frac{{\frac{\text{Ω}^{{(AHL}_{1}+{AHL}_{2}-1)}}{{(AHL}_{1}-1)!{AHL}_{2}!}e}^{-\beta(({(AHL}_{1}-1)+AHL_{2})\varepsilon_{sol}\text{+}\varepsilon_{b_{1}})}}{{{\frac{\text{Ω}^{{(AHL}_{1}+{AHL}_{2})}}{{AHL}_{1}!{AHL}_{2}!}e}^{-(\beta{(AHL}_{1}+{AHL}_{2})\varepsilon_{sol})}+\frac{\text{Ω}^{{(AHL}_{1}+AHL_{2}-1)}}{{(AHL}_{1}-1)!{AHL}_{2}!}e}^{-\beta({(AHL}_{1}-1)+{AHL}_{2})\varepsilon_{sol}\text{+}\varepsilon_{b_{1}})}+{\frac{\text{Ω}^{{(AHL}_{1}+AHL_{2}-1)}}{{AHL}_{1}!{(AHL}_{2}-1)!}e}^{-\beta(\left( {AHL}_{1}+{(AHL}_{2}-1 \right){)\varepsilon}_{sol}\text{+}\varepsilon_{b_{2}})}}$

**(7)**

We could further simplify this equation by multiplying the denominator and numerator by

$$\frac{{AHL}_{1}!{AHL}_{2}!}{\text{Ω}^{{(AHL}_{1}+{AHL}_{2})}};$$

P(${AHL}_{1}$)$=\frac{{\frac{{AHL}_{1}}{\text{Ω}}e}^{-\beta(({(AHL}_{1}-1)+{AHL}_{2})\varepsilon_{sol}\text{+}\varepsilon_{b_{1}})}}{{e^{-(\beta{(AHL}_{1}+AHL_{2})\varepsilon_{sol})}+\frac{{AHL}_{1}}{\text{Ω}}e}^{-\beta({(AHL}_{1}-1)+{AHL}_{2})\varepsilon_{sol}\text{+}\varepsilon_{b_{1}})}+{\frac{{AHL}_{2}}{\text{Ω}}e}^{-\beta(\left( AHL_{1}+{(AHL}_{2}-1 \right){)\varepsilon}_{sol}\text{+}\varepsilon_{b_{2}})}}$

**(8)**

If we consider that the volume of a box is $V_{box}$, then the total volume of the system is $\text{Ω}V_{box}$. The AHL concentration could be written as, $c_{AHL}=\frac{AHL}{\text{Ω}V_{box}}$ . We could also define a local reference concentration which corresponds to having all sites in the lattice occupied as $c_{0}=\frac{1}{V_{box}}$. Hence,

P($c_{{AHL}_{1}}$)$=\frac{\frac{c_{{AHL}_{1}}}{c_{0}}e^{\beta{{(\varepsilon}_{sol}-\varepsilon}_{b_{1}})}}{{1+\frac{c_{{AHL}_{1}}}{c_{0}}e}^{\beta(\varepsilon_{sol}-\varepsilon_{b_{1}})}+\frac{c_{{AHL}_{2}}}{c_{0}}e^{\beta(\varepsilon_{sol}-\varepsilon_{b_{2}})}}$

**(9)**

Analogous equations were derived for the probability of AHL type 2 binding to the receptor. Using the same procedure, we calculate the probability of the unbound AHL as,

P(unbound)$=\frac{1}{{1+\frac{c_{{AHL}_{1}}}{c_{0}}e}^{\beta(\varepsilon_{sol}-\varepsilon_{b_{1}})}+\frac{c_{{AHL}_{2}}}{c_{0}}e^{\beta(\varepsilon_{sol}-\varepsilon_{b_{2}})}}$

**(10)**

| Parameter | Value | References |
| --- | --- | --- |
| $\mu$ | 1.50 (±0.02) hrs^-1^ | (experimentally calculated) |
| S | 10^9^ cells |  |
| *D_AHL_* | 1.764 mm^2^hrs^-1^ | [4] |
| $\rho$ | 2.3 x 10^-9^ nM hrs^-1^ per cell | [5] |
| $\rho_{b}$ | 2.3 x 10^-10^ nM hrs^-1^ per cell | [5] |
| $d_{a}$ | 0.005545 hrs^-1^ | [5] |
| $g$ | 100 at non active state to 600 at activation | [6] |
| $m_{g}$ | 2.5 | [2] |
| $\theta_{g}$ | 70 nM | [2] |
| $T$ | 300K |  |
| C_0_ | 1 M |  |
| $\varepsilon_{sol}-\varepsilon_{lux}={\Delta\varepsilon}_{lux}$ | 12.991 KJ mol^-1^ | [7] |
| $\varepsilon_{sol}-\varepsilon_{las}={\Delta\varepsilon}_{las}$ | 11.426 KJ mol^-1^ | [7] |
| $\varepsilon_{sol}-\varepsilon_{rhl}={\Delta\varepsilon}_{rhl}$ | 9.615 KJ mol^-1^ | [7] |

**Table A. The parameters used in the simulations.** To model the change in g over time, we use a Hill’s function with, g =100 + 500$\frac{\left[ c_{s} \right]^{m_{g}}}{\left[ c_{s} \right]^{m_{g}}+{\theta_{g}}^{m_{g}}}$, so g starts with 100 and increases to 600 at activation. Here C_s_ is the AHL concentration of the senders.


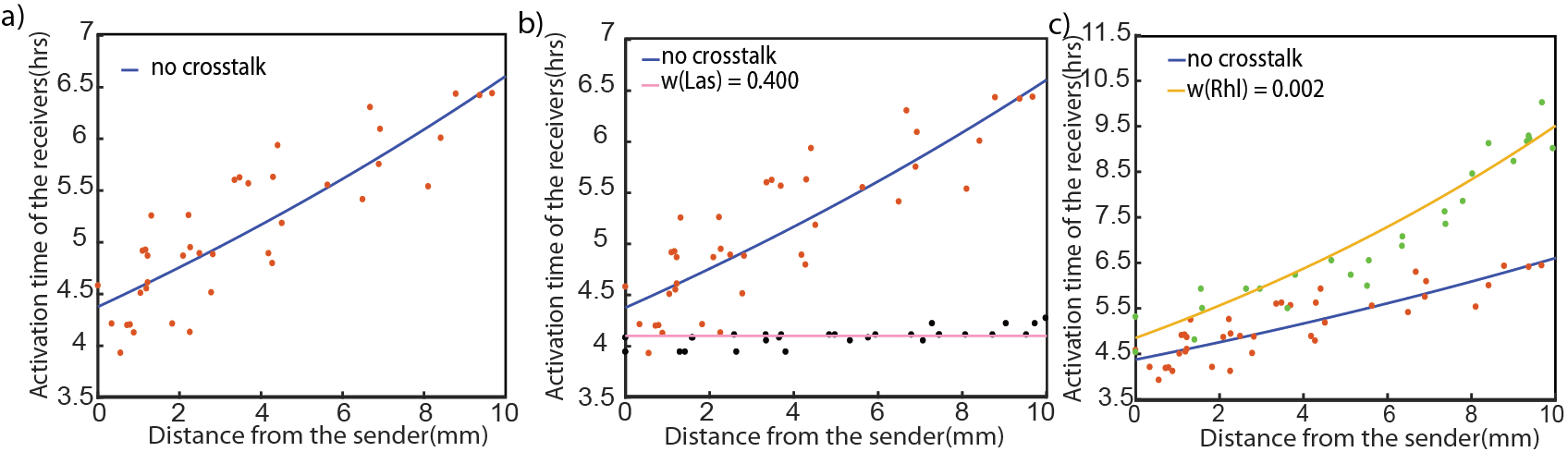


**Figure I.** **Fitting the experimental data to obtain interaction weights.** **A.** The experimental data points in the case of no crosstalk are plotted in red. The blue line shows the simulation result using the best-fit value for the interaction weight. The best fit weight was 0.701± 0.016 for the AHLs produced by the LuxI binding to the LuxR receptor. **B.** Crosstalk with the LasI interactor was fit for the ratio of interactor to receiver level of 0.9. The black data points are from experiments and the pink line shows simulation results using the best-fit interaction weight of 0.400 ± 0.013. **C.** Crosstalk with the RhlI interactor was fit for the ratio of interactor to receiver level of 0.9. The green data points are from experiments and the yellow line shows simulation results using the best-fit interaction weight of 0.002 ± 0.001.


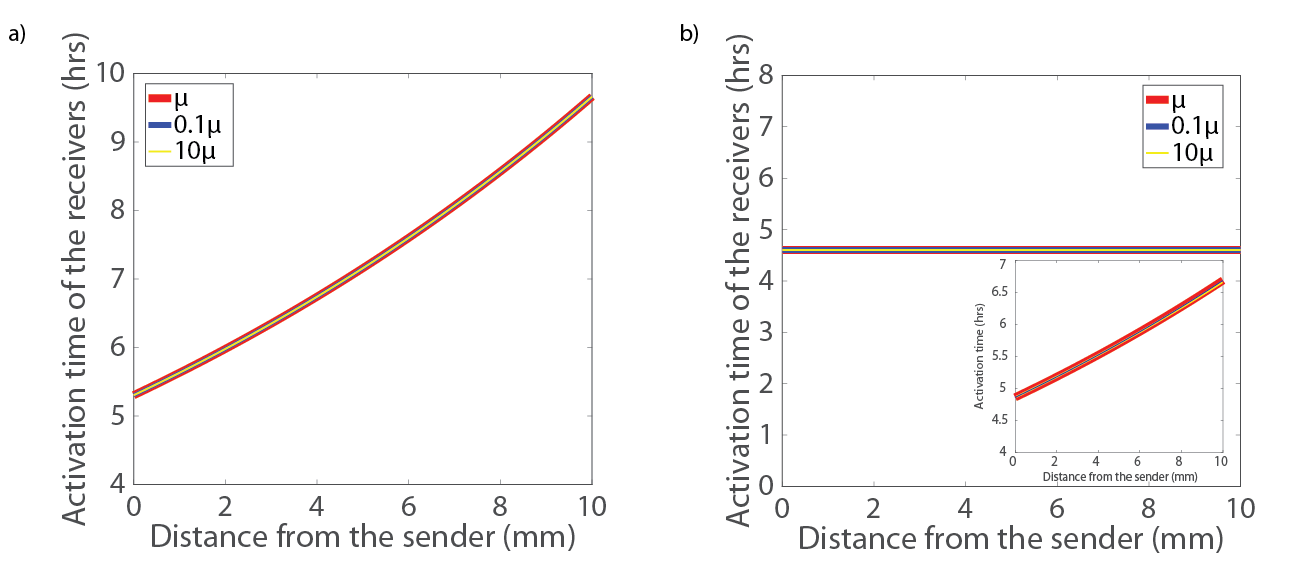


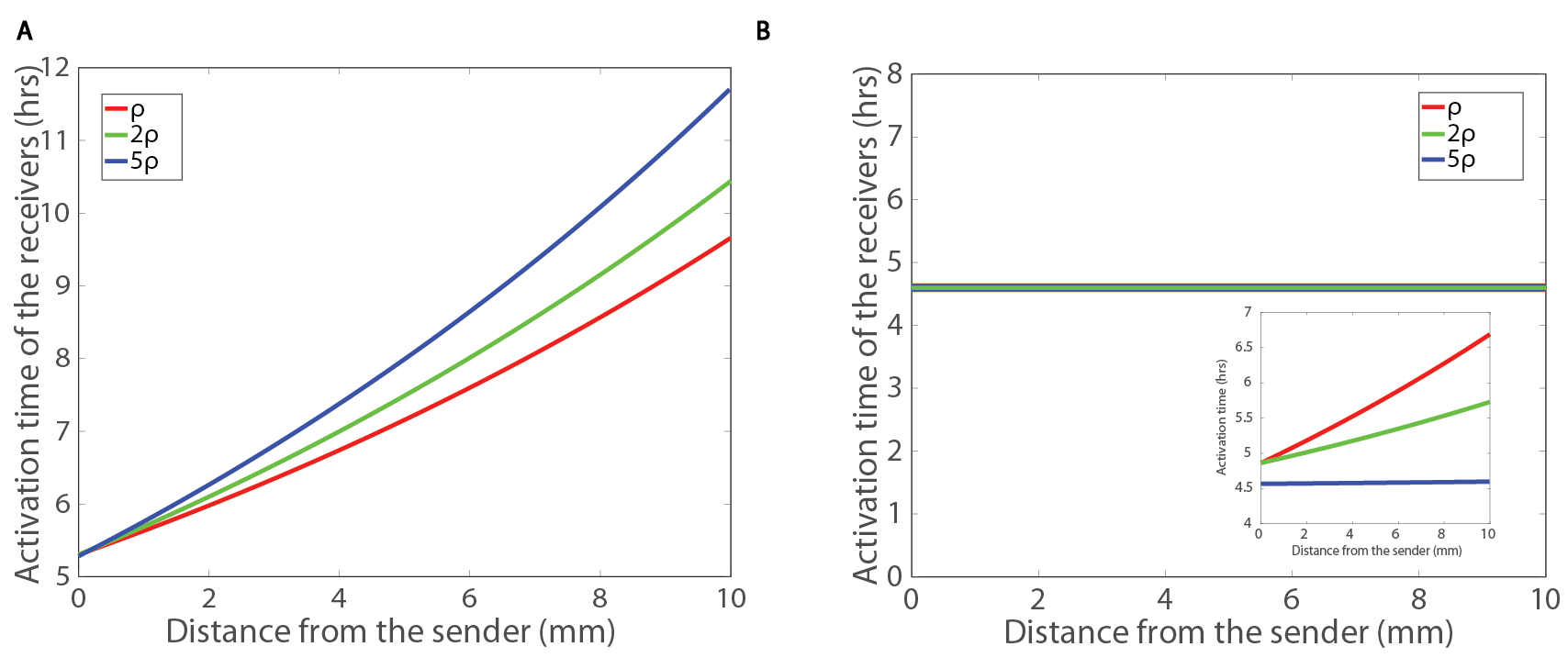
**Figure** **J.** **Testing the effects of the growth rates of the interactors on crosstalk.** Simulations were run using equations from the main text and parameters found in Table 1 and Table A in S1 File. **A.** Activation dynamics when the growth rate of the RhlI interactor is changed by 10 fold at ratio of interactor to receiver of 0.9. **B.** Activation dynamics when the growth rate of the LasI interactor is changed by 10 fold at ratio of interactor to receiver of 0.9. The inset shows results for ratio of interactor to receiver of 0.2. We observe that the crosstalk is not sensitive to the growth rates of the interactors.

**Figure K.** **Testing the effects of the production rates of** **the** **interacting AHLs on crosstalk**. For **A.** the RhlI interactor at 0.9 ratio of interactor to receiver and **B.** the LasI interactor at 0.9 ratio of interactor to receiver. The inset in (B) show results for 0.2 ratio of interactor to receiver. Simulations were run using equations from the main text and parameters found in Table 1 and Table A in S1 File.


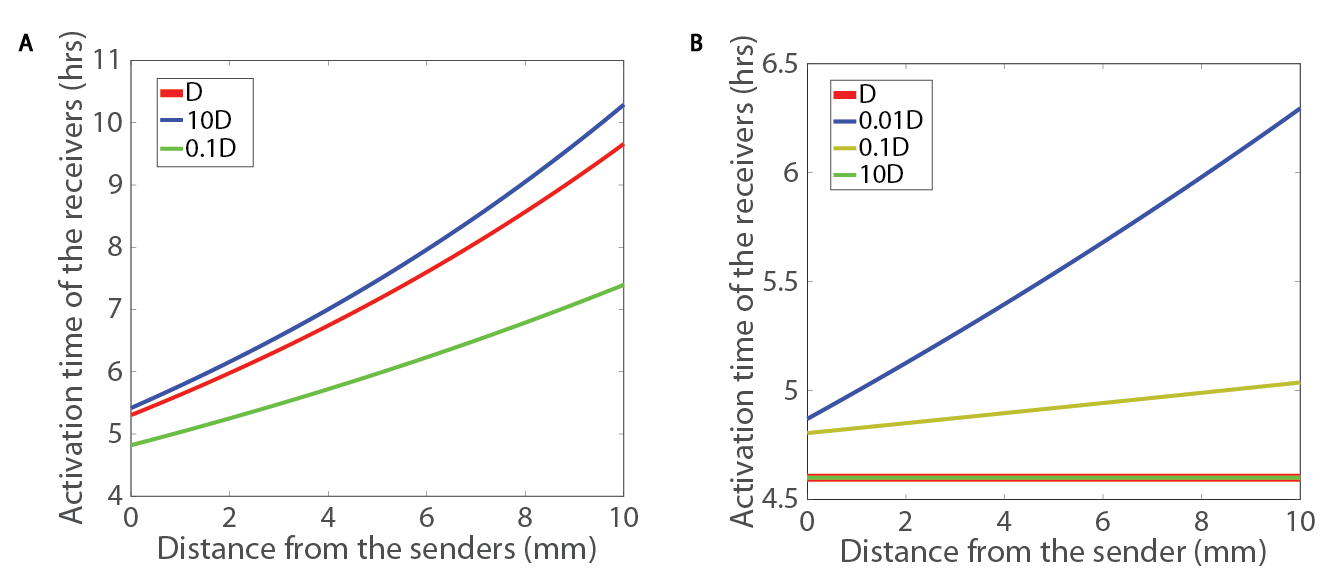


**Figure L.** **Testing the effects of the diffusion coefficient of the AHLs of interactors on crosstalk.** For **A.** RhlI interactor at 0.9 ratio of interactor to receiver and **B.** the LasI interactor at 0.9 ratio of interactor to receiver. Simulations were run using equations from the main text and parameters found in Table 1 and Table A in S1 File. We observe that the crosstalk can be strongly influenced by the diffusion coefficient.


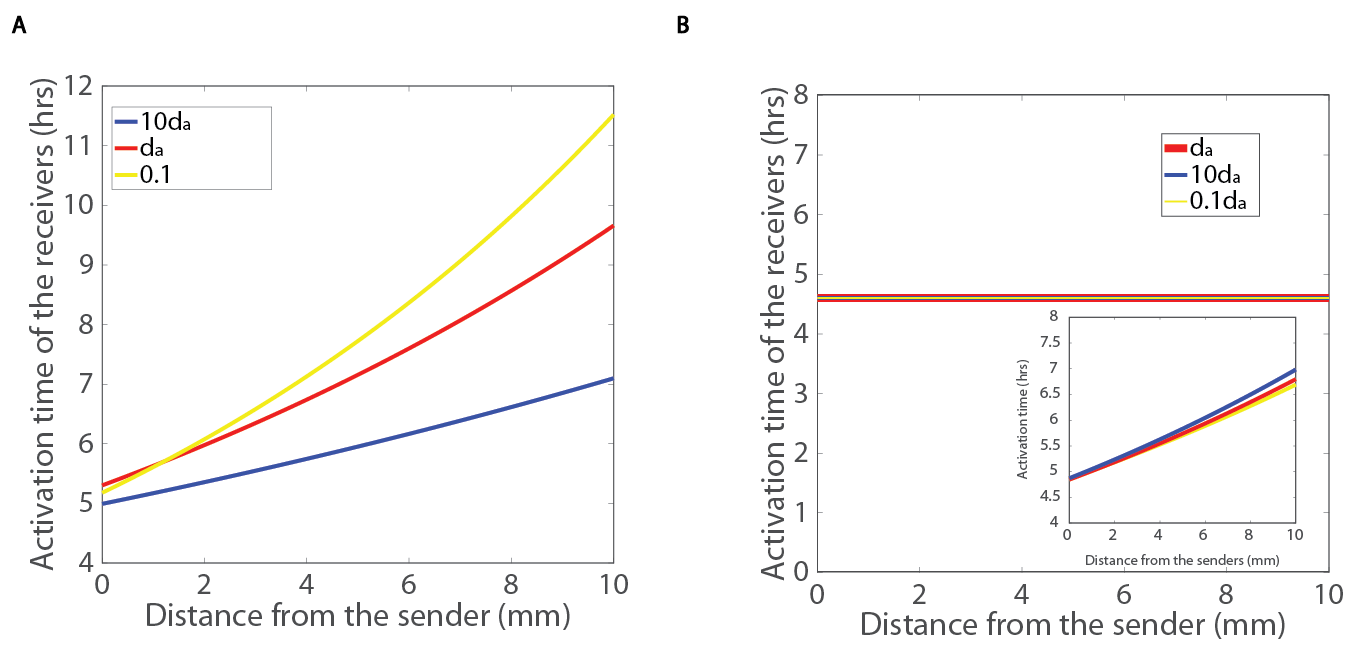


**Figure M.** **Testing the effects of the degradation coefficient of the interacting AHLs on crosstalk.** For **A.** RhlI interactor at 0.9 ratio of interactor to receiver and **B.** the LasI interactor at 0.9 ratio of interactor to receiver. The inset in B is for 0.2 ratio of interactor to receiver. We observe that the crosstalk is sensitive to the degradation coefficient. Simulations were run using equations from the main text and parameters found in Table 1 and Table A in S1 File.


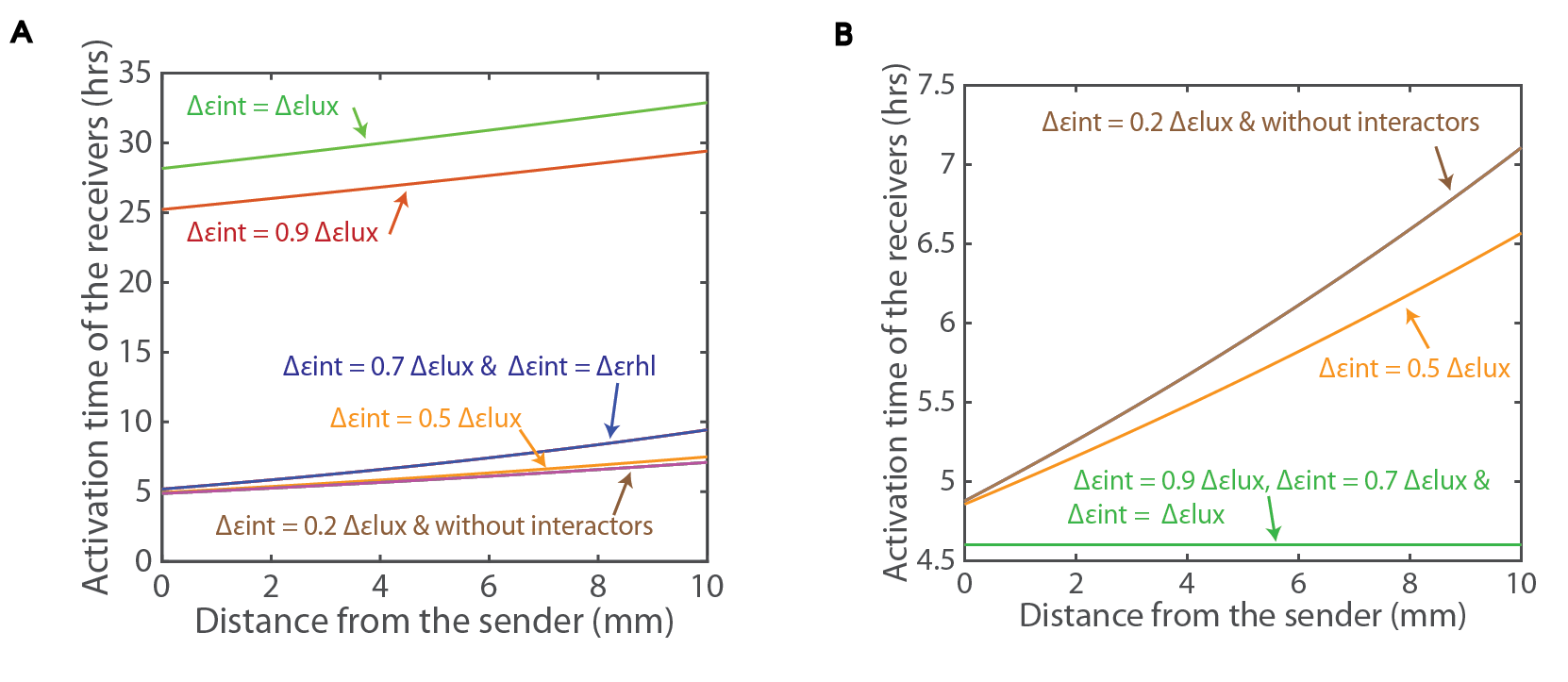


**Figure N.** **The influence of the non-cognate AHL binding energy on crosstalk.** The binding energy of the interactor is${\Delta\varepsilon}_{int}$. In simulations, we tested for various ${\Delta\varepsilon}_{int}$ for the case of **A.** inhibitory crosstalk and **B.** excitatory crosstalk. Here the ratio of interactor to receiver was kept at 0.9.


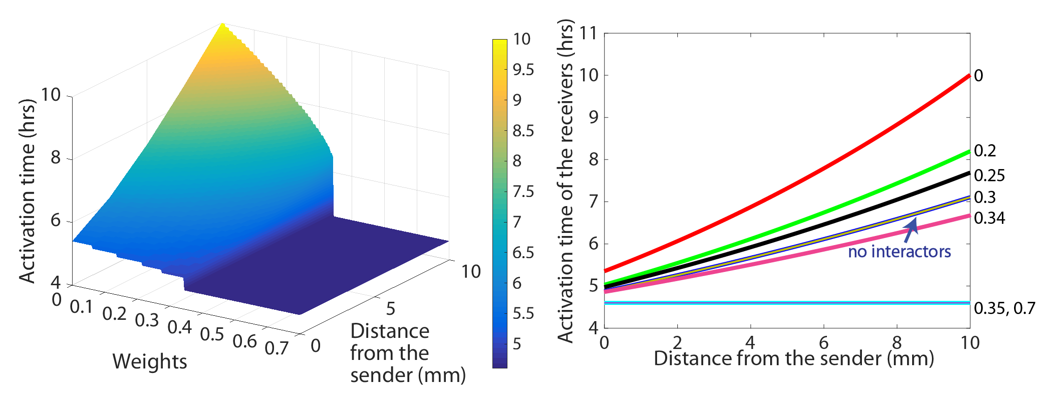
­

**Figure O. The influence of the weights of the AHLs on crosstalk.** The weight parameter of the interactor is changed in simulations for the case of 0.9 ratio of interactor to receiver. In the right, we have the activation time vs. distance projection of the plot on the left for some selected weight values. We observe that at a weight of 0.3 the response of the receivers coincide with the case of no interactors.


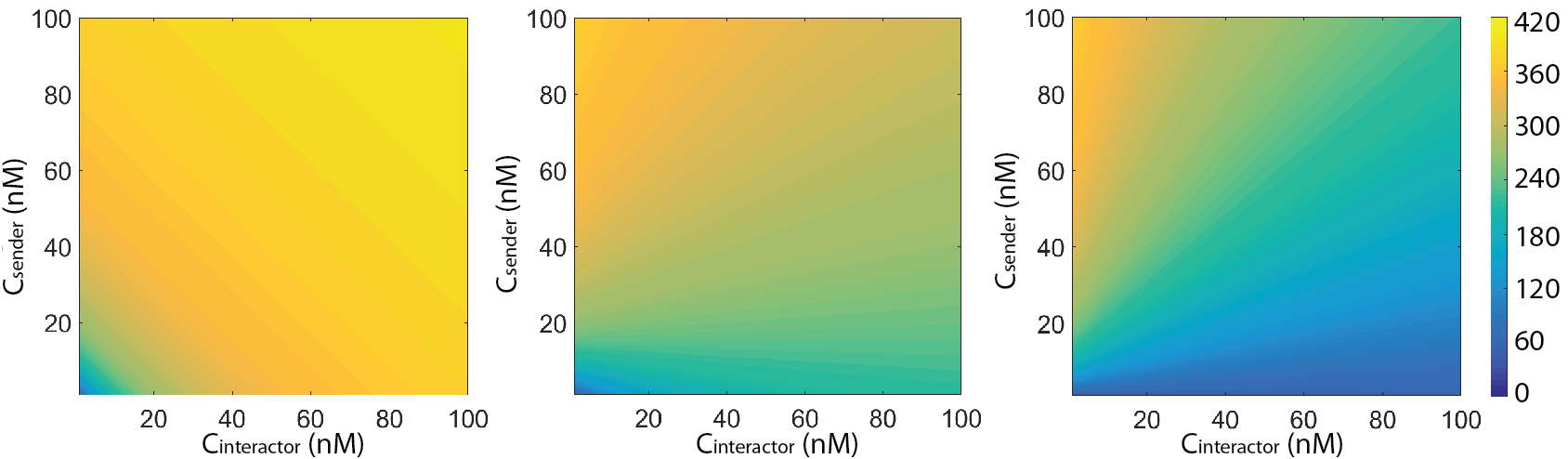


**Figure** **P. Simulated activity profiles of the receivers (per receptor) under well mixed conditions in the presence of signal coming from both a sender strain and an interactor strain.** Plots show the activity of the receiver in the presence of combinations of the sender and interactor signals between 0 and 100 nM. (Left) the interactor strain is the same as the sender, having the LuxI synthase. (Center) the interactor strain has the LasI synthase. (Right) the interactor strain has the RhlR synthase. A_T_= 210 is the threshold level of activity required for activation. The right plot shows a non-zero level of activation even in the case of no sender cells and an inhibitory interactor strain, suggesting that inhibitory crosstalk could be mistaken for excitatory crosstalk in well mixed experiments.


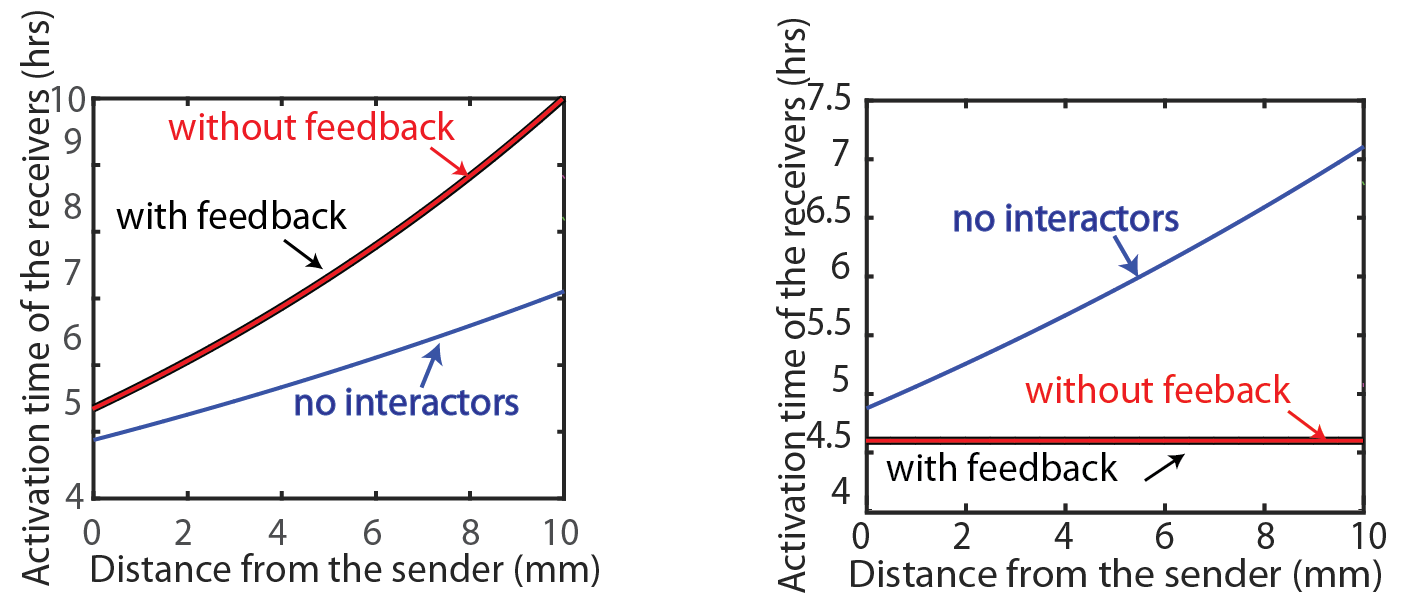
 **Figure Q. The influence of feedback between interactors and senders on activation of the receivers.** Simulation results of when the senders have feedback from the interactor (left=RhlI interactor, right=LasI interactor) compared to when it does not have feedback. To simulate the no feedback condition, the activity of the sender was considered to be only dependent on the sender AHL.


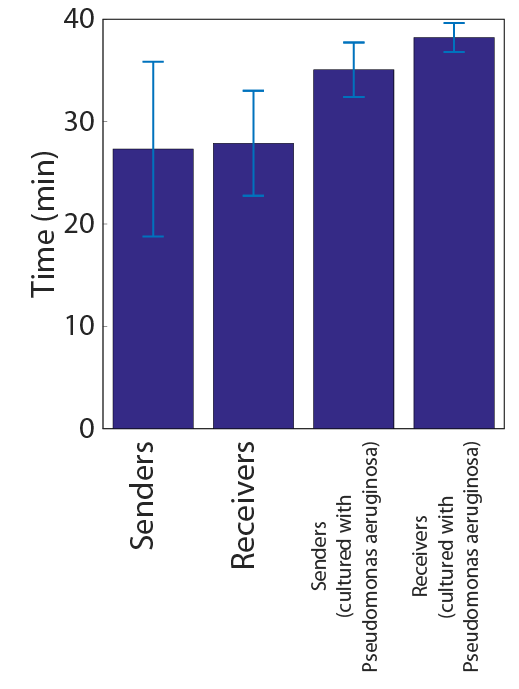


**Figure R. The effect on the growth of the senders and receivers due to *P. aeruginosa.*** The doubling time of the senders (27.3 ± 8.5 min), receivers (27.9 ± 5.1 min), senders cultured with *P. aeruginosa* (35.1 ± 2.7 min), and receivers cultured with *P. aeruginosa* (38.2 ± 1.4 min). The errorbars represents the standard deviation from three sets of replicates.

**Text B. Testing the model for effects of the growth interactions and AHL internalization.**

To test the possible effect of growth influences on the sender-receiver system, caused by the interactor, we considered a competitive Lotka–Volterra model [8,9],

$\frac{\partial n}{\partial t} =\mu n(1-\frac{n_{Total}}{s}$) $-\alpha_{1}n n_{interactor}$ **(11)**

where, $\alpha_{1}$ is the growth effect the interactor species has on the sender-receiver system.

To test the effect of signal internalization we used,

$$\frac{\partial c_{s}}{\partial t}=D_{c}\nabla^{2}c_{s}+ n_{s}(\rho A+\rho_{b})-d_{a}c_{s}-\alpha_{2}n_{int}\frac{\left[ c_{s} \right]^{m_{4}}}{\left[ c_{s} \right]^{m_{4}}+{\theta_{4}}^{m_{4}}}$$

**(12)**

where, $\alpha_{2}$ is the AHL internalizing rate per interactor cell which is modulated by the number of AHLs present in the vicinity of the interactor. Based on [2] we used, $m_{4}$ = 2.5 and $\theta_{4}=70 nM.$


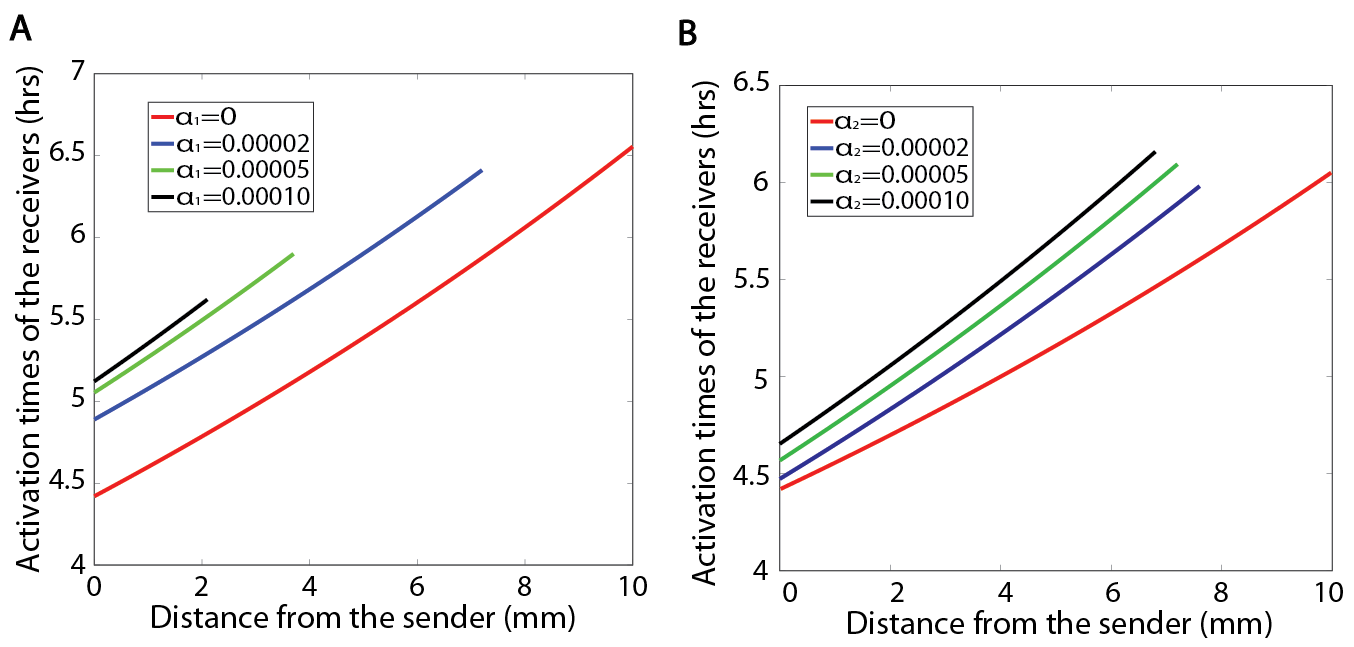


**Figure** **S.** **The impact on crosstalk with non-quorum sensing interactions.** **A.** The interactors have a growth influence on the senders and receivers. Simulations were run using equation (11), based on analysis from [2]. The interactors are at 0.2 ratio of interactor to receiver. **B.** The interactors are capable of internalizing the AHLs produced by the senders. Simulations were run using equation (12), based on analysis from [2].


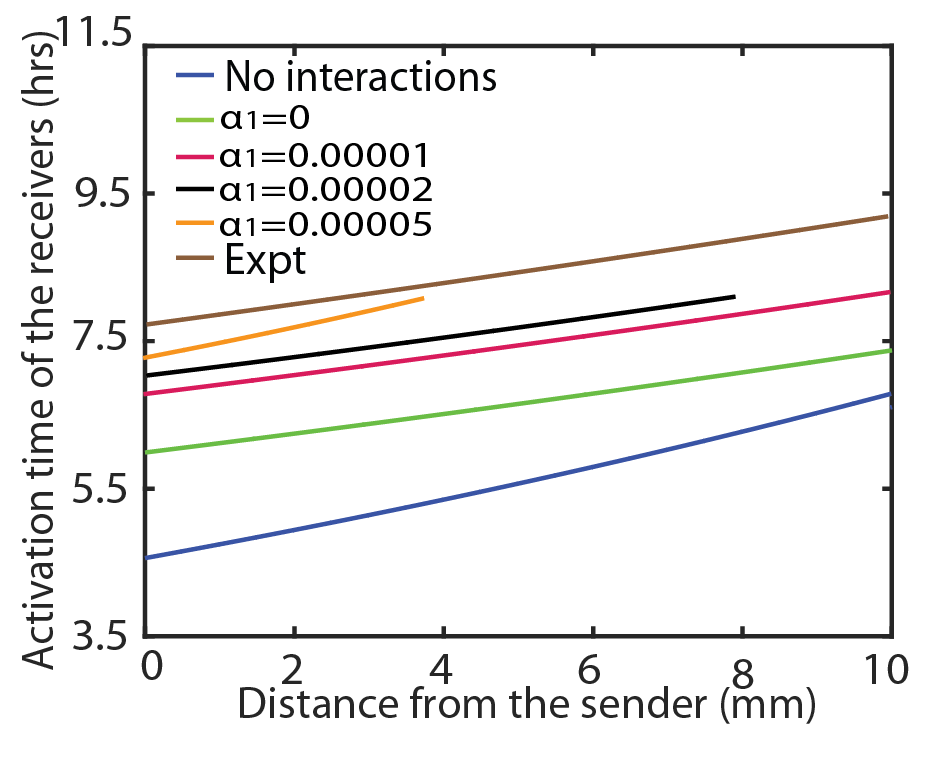


**Figure T. Simulating the impact of *Pseudomonas aeruginosa* growth influences on quorum sensing activation in the receiver strain.** We introduce a growth influence on the receivers and senders, caused by the interactor strain with both *las* and *rhl* systems in simulations. Increasing $\alpha_{1}$ (growth effect parameter), increased the activation times and decreased the activation radius. To yield a similar time shift as observed in experiments (brown) with *Pseudomonas aeruginosa* as the interactor strain, the model would require additional factors such as AHL internalization or non-quorum sensing interspecies regulatory interactions.


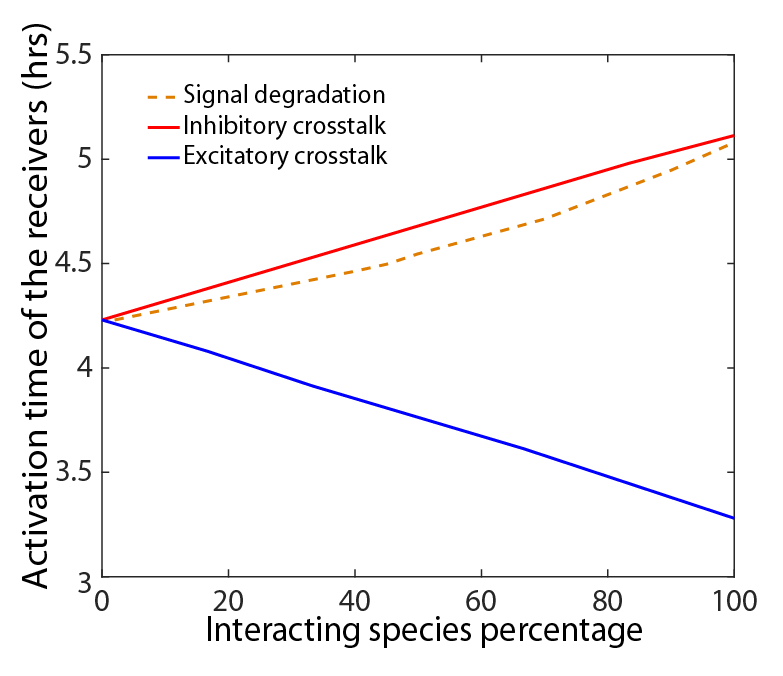
­­

**Figure U. Observing crosstalk and signal degradation in a well-mixed setup.** We simulate a well-mixed system with either inhibitory crosstalk, excitatory crosstalk, or interference by a species producing an AHL degradative enzyme based on [2]. We observe similar responses for the cases of inhibitory crosstalk and signal destruction, indicating complications of distinguishing between these two mechanisms under well mixed conditions.


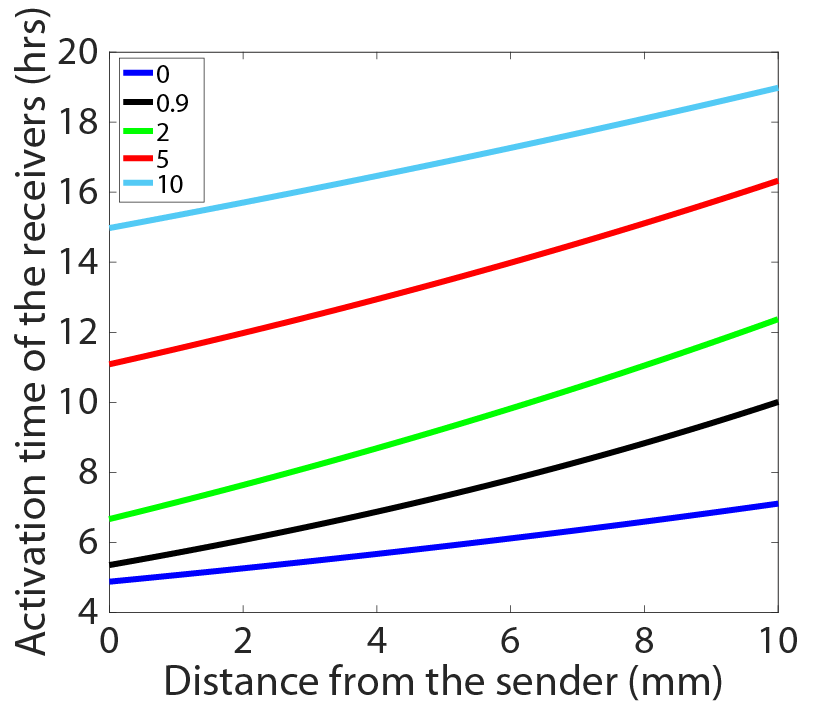


**Figure V. Robustness of the network to interference when there is a large excess of inhibitory interactors.** Simulation data showing the activation time vs. distance from the sender for increasing level of the rhl interactor. Here the lines represent different levels of ratio of interactors to receivers. The activation time of the receivers shifts to around 11-17 hours from 5-7 hours when there is 5 times more interactors than receivers demonstrating ­­­­­diminishing robustness.


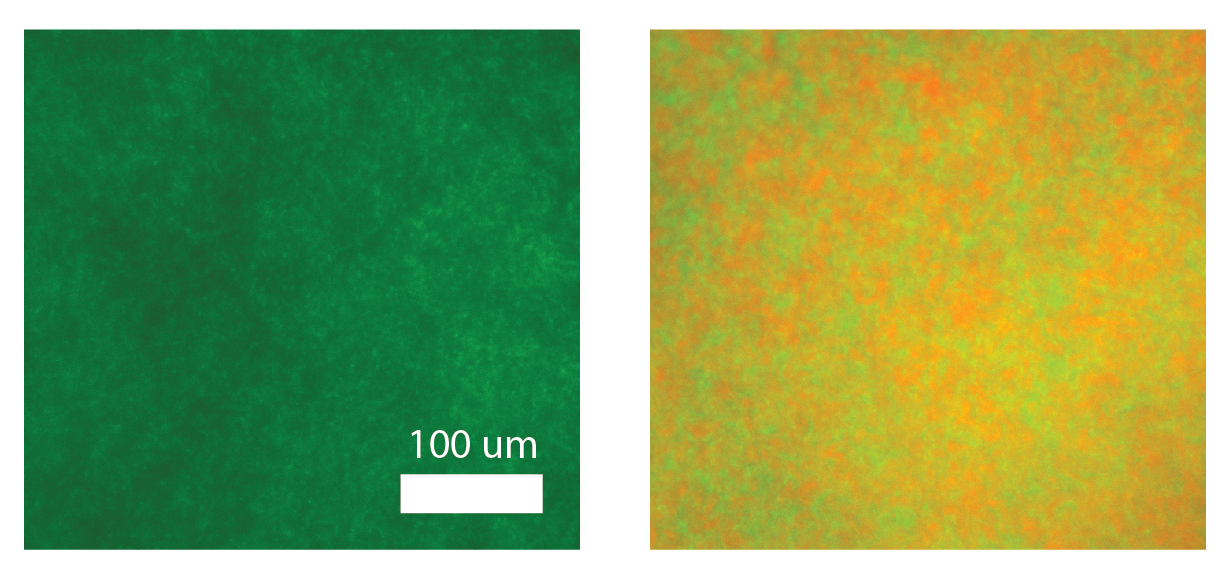


**Figure W. Spatial distribution of cells after 16 h.** Distribution of cells in the assay after 16 hours. Two *E. coli* strains, one producing GFP and the other producing RFP, were uniformly distributed in the assay. Fluorescent images show the distribution of these strains after 16 hours at 37 ^o^C. The left image shows the GFP channel and the right image shows an overlay of the GFP and RFP channels. After growth on the plate, over lengths scales of around 10 microns some regions appear enriched in a single cell type. Over larger lengths, the two cells remaining uniformly mixed.

**References**

1. Danino T, Mondragón-Palomino O, Tsimring L, Hasty J. A synchronized quorum of genetic clocks. Nature. Nature Publishing Group; 2010;463: 326–330. doi:10.1038/nature08753

2. Silva KP, Chellamuthu P, Boedicker JQ. Signal Destruction Tunes the Zone of Activation in Spatially Distributed Signaling Networks. Biophys J. Biophysical Society; 2017;112: 1037–1044. doi:10.1016/j.bpj.2017.01.010

3. Van Valen D, Haataja M, Phillips R. Biochemistry on a leash: The roles of tether length and geometry in signal integration proteins. Biophys J. Biophysical Society; 2009;96: 1275–1292. doi:10.1016/j.bpj.2008.10.052

4. Zengler K, Palsson BO. A road map for the development of community systems (CoSy) biology. Nat Rev Microbiol. Nature Publishing Group; 2012;10: 366–372. doi:10.1038/nrmicro2763

5. Fekete A, Kuttler C, Rothballer M, Hense BA, Fischer D, Buddrus-Schiemann K, et al. Dynamic Regulation of N-Acyl-homoserine Lactone Production and Degradation in Pseudomonas putida IsoF. FEMS Microbiol Ecol. 2010;72. doi:10.1111/j.1574-6941.2009.00828.x

6. Teng S-W, Schaffer JN, Tu KC, Mehta P, Lu W, Ong NP, et al. Active regulation of receptor ratios controls integration of quorum-sensing signals in Vibrio harveyi. Mol Syst Biol. Nature Publishing Group; 2011;7: 491. doi:10.1038/msb.2011.30

7. Gnanendra S, Anusuya S, Natarajan J. Molecular modeling and active site analysis of SdiA homolog, a putative quorum sensor for salmonella typhimurium pathogenecity reveals specific binding patterns of AHL transcriptional regulators. J Mol Model. 2012;18: 4709–4719. doi:10.1007/s00894-012-1469-1

8. Guo X, Boedicker JQ. The Contribution of High-Order Metabolic Interactions to the Global Activity of a Four-Species Microbial Community. PLoS Comput Biol. 2016;12: 1–13. doi:10.1371/journal.pcbi.1005079

9. Bomze IM. Lotka-Volterra equation and replicator dynamics: new issues in classification. 1995;447453.
